# The feedback loop of EFTUD2/c-MYC impedes chemotherapeutic efficacy by enhancing EFTUD2 transcription and stabilizing c-MYC protein in colorectal cancer

**DOI:** 10.1186/s13046-023-02873-0

**Published:** 2024-01-02

**Authors:** Xiaojian Zhu, Changxue Li, Yunfei Gao, Qingyuan Zhang, Tao Wang, Huaixiang Zhou, Fanqin Bu, Jia Chen, Xinjun Mao, Yulong He, Kaiming Wu, Ningning Li, Hongliang Luo

**Affiliations:** 1 Tomas Lindahl Nobel Laureate Laboratory, The Seventh Affiliated Hospital of Sun Yat-sen University, Shenzhen, 518107 China; 2 Digestive Diseases Center, Guangdong Provincial Key Laboratory of Digestive Cancer Research, The Seventh Affiliated Hospital of Sun Yat-sen University, Shenzhen, 518107 China; 3 Department of Otolaryngology, The Seventh Affiliated Hospital of Sun Yat-sen University, Shenzhen, 518107 China; 4 Department of Gastroenterology, Beijing Friendship Hospital, National Clinical Research Center for Digestive Disease, Capital Medical University, Beijing, 100050 China; 5 Department of Anesthesiology, The Affiliated Hospital of Youjiang Medical University for Nationalities, Baise, 533000 China; 6 China-UK Institute for Frontier Science, Shenzhen, 518107 China; 7 Department of General Surgery, The Second Affiliated Hospital of Nanchang University, Nanchang, 330006 China

**Keywords:** c-MYC, Chemotherapy sensitivity, EFTUD2, Prognostic indicator, Ubiquitylation

## Abstract

**Background:**

Chemoresistance presents a significant obstacle in the treatment of colorectal cancer (CRC), yet the molecular basis underlying CRC chemoresistance remains poorly understood, impeding the development of new therapeutic interventions. Elongation factor Tu GTP binding domain containing 2 (EFTUD2) has emerged as a potential oncogenic factor implicated in various cancer types, where it fosters tumor growth and survival. However, its specific role in modulating the sensitivity of CRC cells to chemotherapy is still unclear.

**Methods:**

Public dataset analysis and in-house sample validation were conducted to assess the expression of EFTUD2 in 5-fluorouracil (5-FU) chemotherapy-resistant CRC cells and the potential of EFTUD2 as a prognostic indicator for CRC. Experiments both in vitro, including MTT assay, EdU cell proliferation assay, TUNEL assay, and clone formation assay and in vivo, using cell-derived xenograft models, were performed to elucidate the function of EFTUD2 in sensitivity of CRC cells to 5-FU treatment. The molecular mechanism on the reciprocal regulation between EFTUD2 and the oncogenic transcription factor c-MYC was investigated through molecular docking, ubiquitination assay, chromatin immunoprecipitation (ChIP), dual luciferase reporter assay, and co-immunoprecipitation (Co-IP).

**Results:**

We found that EFTUD2 expression was positively correlated with 5-FU resistance, higher pathological grade, and poor prognosis in CRC patients. We also demonstrated both in vitro and in vivo that knockdown of EFTUD2 sensitized CRC cells to 5-FU treatment, whereas overexpression of EFTUD2 impaired such sensitivity. Mechanistically, we uncovered that EFTUD2 physically interacted with and stabilized c-MYC protein by preventing its ubiquitin-mediated proteasomal degradation. Intriguingly, we found that c-MYC directly bound to the promoter region of EFTUD2 gene, activating its transcription. Leveraging rescue experiments, we further confirmed that the effect of EFTUD2 on 5-FU resistance was dependent on c-MYC stabilization.

**Conclusion:**

Our findings revealed a positive feedback loop involving an EFTUD2/c-MYC axis that hampers the efficacy of 5-FU chemotherapy in CRC cells by increasing EFTUD2 transcription and stabilizing c-MYC oncoprotein. This study highlights the potential of EFTUD2 as a promising therapeutic target to surmount chemotherapy resistance in CRC patients.

**Supplementary Information:**

The online version contains supplementary material available at 10.1186/s13046-023-02873-0.

## Introduction

Colorectal cancer (CRC) ranks among the most prevalent malignancies worldwide [1]. The steady increase in CRC incidence, attributed to lifestyle changes and dietary patterns, has cast a significant shadow on both individual well-being and the socioeconomic landscape [2, 3]. Surgical intervention serves as the primary treatment for early-stage CRC, and it is often considered for patients in intermediate and advanced stages without contraindications, offering the opportunity to excise metastatic lesions [4, 5]. Nonetheless, irrespective of cancer stage, postoperative recurrence and metastasis remain frequent occurrences [6, 7]. Therefore, the administration of adjuvant chemotherapy before and after surgery holds promise for reducing tumor size and curtailing the risk of tumor dissemination. Notably, the emergence of a reversible drug-tolerant persister cell population in CRC, enabling cancer cells to evade chemotherapy and targeted therapies, significantly contributes to treatment failures and tumor relapses. This underscores the pressing imperative for innovative intervention strategies and a simultaneous in-depth investigation into drug resistance mechanisms.

*EFTUD2*, a protein-coding gene encoding a spliceosomal GTPase, plays a pivotal role in splicing precursor mRNAs (pre-mRNAs) into mature mRNAs [8]. Genetic variants in EFTUD2 has been inextricably associated in mandibulofacial dysostosis [9, 10]. Recent research has revealed that elevated EFTUD2 expression is closely linked to tumor growth and poor survival [11]. This association is substantiated by observation that EFTUD2 sustains cancer cell viability and enhances the progression of hepatocellular carcinoma through STAT3 activation [12]. Similarly, Lv Z et al. [13] observed EFTUD2 overexpression in both colon tissue and infiltrating macrophages in a mouse model of colitis-associated cancer. Notably, inhibiting EFTUD2 has been shown to attenuate activation of the NF-KB signaling pathway, leading to reduced production of inflammatory and tumorigenic cytokines. These studies imply that EFTUD2 may have additional roles in modulating cancer development, either independently or dependently of its canonical function in splicing. Nonetheless, whether EFTUD2 is involved in regulating chemoresistance in CRC, along with the precise mechanistic underpinnings, remains elusive.

c-*MYC*, a well-established oncogene, exerts regulatory control over numerous cellular processes, including cell cycle, metabolism, and apoptosis [14]. In CRC, c-MYC is also frequently overexpressed or amplified and confers chemoresistance by modulating drug transporters [15], DNA repair [16], and anti-apoptotic pathways [17].

In this study, we initially observed a notable increase in EFTUD2 expression in 5-FU-resistant CRC cells. Furthermore, we found a significant correlation between elevated EFTUD2 expression and unfavorable prognosis in CRC patients, suggesting its potential as an independent prognostic indicator for predicting patient outcomes. Subsequently, we uncovered an EFTUD2/c-MYC positive feedback loop that enhances c-MYC oncoprotein stability and EFTUD2 transcription, ultimately resulting in impaired efficacy of 5-FU chemotherapy.

## Materials and methods

### Study cohort and design

Between October 2022 and March 2023, we conducted a prospective enrollment of 66 patients who had been diagnosed with CRC and had undergone surgical resection at the Second Affiliated Hospital of Nanchang University (referred to as Nanchang Hospital in this study). The cohort comprised 33 cases of colon adenocarcinoma and 33 cases of rectal adenocarcinoma. Prior to their surgeries, none of the patients had received any form of treatment, including chemotherapy, radiotherapy, or biotherapy. Pathological examination of all specimens confirmed the diagnosis of adenocarcinoma. Surgically resected tissue samples were promptly preserved as backups in an ultra-low temperature refrigerator at -80 °C. Informed consent was obtained from each patient before their surgery, following approval by the Ethics Committee of Nanchang Hospital (Ethics *No.2023.034*). Patients who met emergency surgery criteria (e.g., acute bowel obstruction, tumor perforation), those with postoperative pathology suggesting positive cut margins, and those with incomplete case information were excluded from the study.

### Cell culture and cell line construction

The human CRC cell lines SW620 (RRID: CVCL_0547), SW480 (RRID: CVCL_0546), HCT116 (RRID: CVCL_0291), Caco-2 (RRID: CVCL_0025), LoVo (RRID: CVCL_0399), and the normal colon epithelial cell line NCM460 (RRID: CVCL_0460) were all purchased from the Cell Bank of the Chinese Academy of Sciences. Upon obtaining the cell lines, immediate validation procedures were performed, including recent analysis involving short tandem repeat (STR) analysis, mycoplasma testing, and cell viability assessment. In brief, the validation of all cell lines was conducted using the LONZA MycoAlertTM mycoplasma detection kit (Lonza, #LT07-418), with all results yielding negative outcomes. Additionally, none of the cell lines used were found in the commonly misidentified cell line database maintained by the International Cell Line Authentication Committee.

All cell lines were cultured in Dulbecco’s Modified Eagle’s Medium (DMEM; Gibco, #11965092) or Roswell Park Memorial Institute 1640 (RPMI 1640; Gibco, # 11875119) supplemented with 10% fetal bovine serum (FBS; Gibco, #10099-141) in a humidified incubator at 37 °C with 5% CO_2_.

To establish EFTUD2-overexpressing SW480 cell lines, a lentiviral vector carrying EFTUD2 (pcDNA3.1-Flag-EFTUD2-GFP/Puro) was transfected into the cells, while an empty vector (pcDNA3.1-Flag) was used as a negative control. The cloning primers used for EFTUD2 overexpression were as follows:

- Forward: 5′-TAACCTCTGAAAGAGGAACTTGGTTAGGTACCATGTGTCAGACACTTGCTCAGTCT-3′,
- Reverse: 5′-TCGTACACCTTGGAAGCCATGGTGGCTAGCGATGCTCTCGCCTGCTCAGCT-3′.

Similarly, to knock down EFTUD2 expression, lentiviral-mediated EFTUD2-shRNA was transfected into the HCT116 and Caco-2 cell lines using specific fragments, with a control group established for comparative purposes. The sequence of EFTUD2 shRNAs were as follows:

- shRNA#1: 5′-CCCATTATTAAGCCAGTGAAA-3′.
- shRNA#2: 5′-GCCTCTCACAGAACCCATTAT-3′.

Following infection, stable cell lines were generated by passaging cells for 72 h in a medium containing 5 µg/mL puromycin (Sigma, Missouri, USA, #S250) until all uninfected cells were eliminated. The transfection efficiency was assessed using both Western blotting and RT-qPCR.

### MTT assay

Briefly, stably-transfected CRC cells were seeded into 96-well plates (Thermo Scientific, #60180-P210) at a density of 2000 cells per well. The cells were then incubated in a cell culture incubator until a confluent monolayer formed at the bottom of each well. Subsequently, a gradient of 5-fluorouracil (5-FU; Abmole Bioscience, Shanghai, China, #51-21-8) was added, with five gradients of 100 µL per well, and three replicate wells for each gradient were established. After a 24-hour incubation, the cells were observed under an inverted microscope. Following this, 20 µL of MTT solution (5 mg/mL, 0.5% MTT; Invitrogen, #M6494) was added to each well, and the cells were further incubated for 4 h. Afterward, the cells were washed twice with phosphate-buffered saline (PBS; Gibco, #10010031) and the culture solution containing MTT was added. The cells were then incubated until formazan crystals formed. Subsequently, the culture solution was aspirated, and 150 µL of dimethyl sulfoxide (DMSO; Invitrogen, #D12345) was added to each well to fully dissolve the formazan crystals. The absorbance value of each well was measured at 490 nm using an enzyme-linked immunosorbent assay reader.

### Immunohistochemistry (IHC) staining

 The embedded paraffin tissue sections (5 µM) were initially parched in a 65 °C oven for 30 min to permit water evaporation and paraffin melting, ensuring firm adhesion of tissue sections to the slides. Subsequently, the sections were infiltrated with pre-warmed closure permeabilization solution (40 mL PBS + 120 µL TritonX-100 + 400 µL 30% H_2_O_2_) for 30 min to suppress endogenous peroxidase activity. Antibody incubation was carried out using EFTUD2 primary antibody (Proteintech, #10208-1-AP, 1:150) after blocking with goat serum to diminish the non-specific background staining. This was followed by incubation with HRP-conjugated goat anti-rabbit IgG secondary antibody (ZSGB-BIO, PV-6001). Finally, the paraffin sections were visualized with 3, 3-diaminobenzidine tetrahydrochloride (DAB; Sigma, #D5637). Further experimental details can be found in our previous literature with more specificity [18].

### Immunofluorescence (IF) staining

In the study, CRC samples underwent fixation using 4% paraformaldehyde (Thermo Scientific, #FB002) and were subsequently embedded in Tissue-Tek O.C.T. compound (Sakura Finetek USA, Torrance, CA). Following this, 3 mm frozen sections underwent a blocking step with 3% donkey serum albumin in phosphate-buffered saline (PBS; Gibco, #10010031) for 30 min at 37 °C. For immunostaining, an anti-EFTUD2 rabbit polyclonal antibody (Proteintech, #10208-1-AP, 1:200) was diluted in PBS and incubated overnight at 4 °C. Subsequently, a goat anti-rabbit IgG Fluor Plus 647 secondary antibody (Thermo Scientific, 1:500, #A32733) was applied at room temperature for 1 h. Sections were then covered with Vectashield mounting medium containing 4’,6-diamidino-2-phenylindole (DAPI; Abcam, #ab104139). The obtained images were observed using a Confocal Laser Microscope System (Zeiss, LSM900), and the percentages were quantified using ImageJ software.

Simultaneously, cell samples (3000 cells per well) were fixed with 4% paraformaldehyde for 30 min and permeabilized with 0.1% Triton X-100 (Sigma, #T8787-100ML) for 15 min. After blocking with 3% donkey serum albumin, cells were incubated with primary mouse antibodies against EFTUD2 (Proteintech, #67855-1-Ig, 1:500) and primary rabbit antibodies against c-MYC (Proteintech, #10828-1-AP, 1:250) at 4 °C overnight. Subsequent staining involved goat anti-rabbit IgG Fluor 488 secondary antibody (Thermo Scientific, 1:500, #A11034) and goat anti-mouse IgG Fluor 568 secondary antibody (Thermo Scientific, 1:500, #A11004) at room temperature for 1 h. Nuclei were counterstained with DAPI, and images were acquired using a Confocal Laser Microscope System (Zeiss, LSM900).

### Western blotting

To extract total protein from harvested CRC cells, Pierce IP lysis buffer (Thermo Scientific, #87788) was employed. The protein concentration was determined via the BCA method (Thermo Scientific, #23227) before adding protein buffer and boiling the samples for 10 min. Next, electrophoresis was performed using 10% SDS-PAGE gels (Solarbio, #P1200-50T), and the proteins were transferred to a PVDF membrane (Thermo Scientific, #88518). The membrane was then sealed with 5% skimmed milk at room temperature for 1 h, followed by overnight incubation at 4 °C with the appropriate primary antibodies: EFTUD2 (Proteintech, #67855-1-Ig, 1:2000), c-MYC (Proteintech, #10828-1-AP, 1:4000), Flag (Abcam, #ab236777, 1:500) and β-Actin (Proteintech, #81115-1-RR, 1:5000). Subsequently, the membrane was washed 3 times with 1× TBST (Thermo Scientific, #28360) for 10 min each wash, and then incubated with the appropriate secondary antibody, including HRP-conjugated goat anti-rabbit IgG (Proteintech, #PR30011, 1:5000) and HRP-conjugated goat anti-mouse IgG (BOSTER, #BA1050, 1:5000), for 1 h at room temperature. Finally, the luminescence signal was visualized using ECL (Solarbio, #PE0020), and the data were analyzed using ImageJ software.

### Real-time quantitative PCR (RT-qPCR)

To perform RNA extraction, 80 mg of frozen CRC tissue samples were ground, or 2 × 10^6^ CRC cells were collected. The two types of samples were then subjected to total RNA extraction using the TRIzol method (1 mL; TRIzol Reagent, #15596026) and a total RNA isolation kit (Vazyme, #RC112-01), respectively. The extracted RNA underwent quality assessment by measuring purity, concentration, and integrity, aiming for an A260/A280 ratio between 1.8 and 2.0. Subsequently, reverse transcription for cDNA synthesis was carried out using mRNA as the template, facilitated by a QIAGEN kit (#208156). For PCR amplification, the cDNA generated earlier was employed as the template, and target gene-specific primers for *EFTUD2* were utilized: Forward (5′-GAAGCACGATCTCCGGCA-3′) and Reverse (5′-AGATCTTGGCAAAGGAGCCC-3′). To explore the metastatic potential of the EFTUD2/c-MYC axis, target gene-specific primers for epithelial-mesenchymal transition (EMT) markers were utilized:

- *Vimentin*: Forward (5′-AAGACGGTTGAAACTAGAGATGGAC-3′) and Reverse (5′-TTGCTGGTAATATATTGCTGCACTGA-3′),
- *E-cadherin*: Forward (5′-GAGTGCCAACTGGACCATTCAGTA-3′) and Reverse (5′-CACAGTCACACACGCTGACCTCTA-3′),
- *ZEB1*: Forward (5′-TTACACCTTTGCATACAGAACCC-3′) and Reverse (5′-TTTACGATTACACCCAGACTGC-3′),
- *Slug*: Forward (5′-GCTACCCAATGGCCTCTCTC-3′) and Reverse (5′-CTTCAATGGCATGGGGGTCT-3′).

Finally, data analysis involved the application of the 2^−ΔΔCT^ method to calculate mRNA relative expression levels. The expression of the target gene was normalized to *GAPDH*: Forward (5′-GGTCACCAGGGCTGCTTTTA-3′) and Reverse (5′-CCCGTTCTCAGCCATGTAGT-3′) used as reference genes.

### EdU cell proliferation assay

Cells were initially plated at a density of 4 × 10^4^ cells/well in a 96-well plate and cultured in medium containing 5-FU (25 µM). After 24 h, a 50 µM EdU medium was prepared by diluting the EdU solution (Invitrogen, #A10044) in cell culture medium at a ratio of 1:1000. Subsequently, 100 µL of this diluted EdU medium was added to each experimental group and incubated for 2 h in a standard cell culture incubator. After incubation, the EdU-containing medium was carefully removed, and cells were washed twice with PBS for 5 min each to remove unincorporated EdU. Following this, cells were fixed with 4% paraformaldehyde (Thermo Scientific, #FB002), subjected to Apollo staining for EdU labeling, and stained with Hoechst 33342 for DNA. Finally, images were captured, and EdU-positive cells, indicative of cell proliferation, were quantified in the obtained images using ImageJ software.

### Clone formation assay

Cell lines in good condition were digested using 0.25% trypsin. 500 cells/well were inoculated in 6-well plates (Thermo Scientific, #140675), dispersed evenly and incubated with 5-FU (25 µM) for 14 days in a 37 °C, 5% CO_2_ incubator. After the incubation period, the cells were washed twice with PBS. Subsequently, the cells were fixed with 4% paraformaldehyde (Thermo Scientific, #FB002) for 30 min, stained with crystal violet (Thermo Scientific, #R40052) staining solution for 30 min, rinsed to remove excess the staining solution with running water, and allowed to dry. The flat dish was inverted, and a transparent film with a grid was superimposed. Cell clones were counted directly, and the clone formation rate was calculated as (number of clones/inoculated cells) × 100%.

### TUNEL apoptosis staining

Apoptotic cells were detected using a TUNEL assay kit (Solarbio, #T2195) according to the manufacturer’s protocol. Briefly, cells were fixed with 4% paraformaldehyde at 37 °C for 15 min and then blocked using blocking buffer. Subsequently, the cells were permeabilized with 0.1% Triton X-100 in 0.1% sodium citrate for 2 min on ice. The cells were incubated with TUNEL reaction mixture for 1 h at 37 °C. DAPI was used to counterstain the nuclei, and the numbers of TUNEL-positive cells were recorded under a fluorescence microscope.

### Animal in vivo experiments

The CRC cell lines SW480 and HCT116 were cultured in DMEM medium supplemented with 10% FBS. After trypsin digestion, the cells were combined with 50% Matrigel (BD, #354234) to achieve a final concentration of 5 × 10^6^ cells/200 µL. To establish a xenograft mouse model of CRC, commonly known as a cell-derived xenograft or CDX model, a cell suspension from SW480 and HCT116 was subcutaneously injected into the axilla region of 40 five-week-old BALB/c nude mice. These mice were procured from SJA Laboratory Animal Co., Ltd. (Hunan, China), and were acclimated to specific pathogen-free (SPF) conditions for one week prior to the experiment. The animal experimentation in this study was conducted in compliance with ethical standards and received approval from the Ethics Committee of Nanchang Hospital (Ethics No. 2023.049), adhering to institutional guidelines and regulations. When the tumors had grown to approximately 100 mm^3^ in size, intraperitoneal injections of 5-FU (30 mg/kg) were administered every 2 days until the conclusion of the experimental tumor harvesting period. Tumor size and volume were monitored using an in vivo imaging system (IVIS) (Newton 7.0, France). The tumors were surgically excised for subsequent analysis and research purposes.

### Chromatin immunoprecipitation (ChIP)

The CRC cell line was cultured under standard conditions of 37 °C with 5% CO_2_ until it reached a density of 70–80%. To initiate cross-lining, 1% formaldehyde was applied for 10 min, and the reaction was quenched with 0.125 M glycine. Subsequently, the cells were lysed using ChIP lysis buffer supplemented with a protease inhibitor cocktail (Thermo Scientific, #87786). The lysis was sonicated, and then incubated with protein A/G magnetic beads (Solarbio, #M2400), 10 µg of c-MYC antibody (Proteintech, #10828-1-AP), or 10 µg rabbit IgG antibody (Proteintech, #30000-0-AP) as a negative control. The pulled-down and purified DNA fragments were subjected to qPCR analysis using the specified primers (Forward: 5′-cctagcaactgcgctaacag-3′, Reverse: 5′-aggaccagttccgaggtatg-3′). Relative enrichment was calculated as the amount of amplified DNA normalized to the input.

### Dual luciferase reporter assay

In this experiment, 0.8 µg of the psiCHECK vector expressing either the wild-type (WT) or the mutant EFTUD2-promoter, along with pcDNA3.1 vector expressing either c-MYC gene or c-MYC shRNA, was transfected into HCT116 and SW480 cells (4 × 10^4^) in each well of 24-well plates using Lipofectamine 3000 (Invitrogen, #L3000015). Additionally, 5 ng of Renilla vectors (pRL-TK) were co-transfected as an internal control. After 48 h of transfection, luciferase activity was determined using a dual luciferase reporter assay kit (Promega, #E1910) on a high sensitivity tube luminescence detector (BLT Lux-T020, China). Renilla activity in each well was used for normalization. The critical sequences used in the luciferase reporter assay are detailed in Supplementary Table 3.

### Protein half-life assay

Cells were exposed to 20 µg/mL cycloheximide (CHX) (Medchem Express, #66-81-9), effectively inhibiting the synthesis of new proteins. Following this CHX treatment, the cells were collected at defined time points (0, 30, 60, 120 min), and protein levels were evaluated by Western blotting.

### Ubiquitination assay

Ubiquitination assay was performed according to the instructions provided with the ubiquitination kit (Invitrogen, #F20650). Briefly, Flag-EFTUD2 or sh-EFTUD2 plasmids were transfected into CRC cells that had reached approximately 80% confluency. Twenty-four hours post-transfection, 10 µM MG132 (Medchem Express, #133407-82-6) was added to culture medium, and cells were incubated for 6 h. Subsequently, cells were lysed using a lysis buffer for immunoprecipitation (Thermo Scientific, #87787). The supernatant was incubated with an anti-c-MYC antibody (Proteintech, #10828-1-AP) overnight at 4 °C, and subsequently with agarose A/G beads (Solarbio, #M2400) for 4–6 h. Finally, the eluted proteins were detected by immunoblotting using a ubiquitin antibody (Proteintech, #10201-2-AP, 1:1000) to detect the extent of ubiquitination modifications of c-MYC.

### Co-immunoprecipitation (Co-IP)

Precooled lysis buffer and PMSF (Thermo Scientific, #36978, 100:1) were used to lyse SW480 and HCT116 cells for 10 min. The supernatant was incubated with 2 µg of EFTUD2 antibody (Proteintech, #10208-1-AP), c-MYC antibody (Proteintech, #10,828-1-AP), Flag antibody (Abcam, #ab236777), and V5 antibody (Abcam, #ab309485) for 1 h. Following this incubation, 40 µL of protein A/G PLUS-Agarose (Santa Cruz, #20423) beads were added. After an overnight incubation at 4 °C, the beads were washed four times with immunoprecipitation buffer. Finally, the precipitate was dissolved in 40 µL of 1× electrophoresis sample buffer, boiled for 10 min, and subsequently subjected to Western blotting using specific antibodies.

### Analysis of public databases, published datasets, and in-house data

The transcriptome RNA-seq data and corresponding clinical information for colon adenocarcinoma (COAD) and rectum adenocarcinoma (READ) were retrieved from The Cancer Genome Atlas (TCGA) and Gene Expression Omnibus (GEO) database [19] across three datasets (GSE166900, GSE81005, GSE81008). The analysis was conducted using the R/Bioconductor package. Differential gene screening criteria were set at *P*. Adjust < 0.05 and |Log2 Fold Change| ≥ 1.

The proteomic data were acquired from Clinical Proteomic Tumor Analysis Consortium (CPTAC) [20]. The drug response data were obtained from the Cancer Therapeutics Response Portal (CTRP) [21] and RNAactDrug [22]. The protein interaction data was sourced from the EMBL-EBI database [23]. The transcriptome data related to c-MYC knockdown in HCT116 cells were retrieved from the KnockTF database [24]. Single-cell transcriptomic analysis and visualization were performed using the GRNdb online database [25] to reveal gene regulatory relationships.

The Cistrome Data Browser [26], housing ChIP-seq data, and the JASPAR database [27] were used to identify and analyze potential c-MYC binding motifs within the promoter region of EFTUD2. MYC binding peaks were visualized through the UCSC hg38 genome Browser [28]. The promoter sequences of EFTUD2 were obtained from NCBI.

For molecular docking analysis, two protein structures were initially submitted to HDOCK (hdock.phys.hust.edu.cn) to perform the docking simulation and obtain the output poses. Once the most promising binding poses or complexes were selected, LigPlot + 2.2.4 was utilized to analyzed the protein-protein interactions in the chosen complexes, illustrating functional residues participating in hydrogen bonding, salt bridges, and hydrophobic contacts. The protein-protein docking conformation was visualized using PyMol 2.2.0.

Receiver operating characteristic curves (ROC) analyses were performed using patient survival status and EFTUD2 expression data. The data consisted of three datasets: CRC (TCGA train set, RNA-seq, *n* = 698), CRC (TCGA validation set, RNA-seq, *n* = 698), and CRC (TCGA test set, RNA-seq, *n* = 742). Furthermore, clinical prognostic data and EFTUD2 mRNA expression data from Nanchang Hospital (*n* = 66) were used for validation via ROC analysis. The pROC v1.18.0 package was used to conduct the ROC analysis and ggplot2 was employed to create visual representations of the results.

Proportional hazards regression models were applied for univariate and multivariate analysis of prognostic data using R software (v3.3.1) and its associated ‘survminer’ and ‘survival’ packages.

Gene set enrichment analysis (GSEA) was carried out using GSEA tool v3.0 [29], with the input gene sets sourced from gene sets collections, including Hallmarks, C5, and C7 from MsigDB v6.2. Enrichment scores were calculated through analyses performed on the GenePattern platform, employing the classical GSEA method. The results of the enrichment analysis were visualized using ggplot2.

### Statistical analysis

All values were presented as means ± standard deviation (SD) and statistical analyses and visualization were conducted using GraphPad Prism9.0 (GraphPad Prism Software, USA). Student’s t-test and Mann-Whitney U-test were used to determine statistical differences between two groups. One-way ANOVA was used to determine statistical differences between multiple testing. The chi-square test was used to analyze the relationship between EFTUD2 expression and clinicopathological characteristics. The survival analysis was performed using the Kaplan-Meier technique and Cox regression analysis. Each experiment was performed three times. *P*-values < 0.05 were considered statistically significant and labelled as follows: ns stands for not significant, *P* > 0.05, *P < 0.05, **P < 0.01, ***P < 0.001, and ****P < 0.0001.

## Results

### Bioinformatics analysis identified that EFTUD2 was upregulated in 5-FU-resistant CRC cells

Chemotherapy resistance remains a pressing challenge in the treatment of CRC, necessitating the exploration of novel therapeutic approaches to enhance treatment efficacy [30, 31]. To address this issue, we conducted a comprehensive transcriptional analysis by comparing parental and 5-FU resistant cells across three GEO datasets (GSE166900, GSE81005, GSE81008). This analysis unveiled 18 significantly upregulated genes shared among CRC chemoresistant cells (Fig. S1A and B). Among these genes, we employed multivariate Cox regression analysis and identified four genes (*EFTUD2*, *VIM*, *EFNB2*, *PLAC1*), with *EFTUD2* emerging as the top candidate, that independently predicted overall survival (OS) in CRC patients (Fig. S1C). Subsequently, we harnessed the RNAactDrug database to investigate the correlation between the EFTUD2 expression and common chemotherapy regimens used in CRC clinical practice, including FOLFOX (Calcium Folinate, Fluorouracil, Oxaliplatin) and FOLFIRI (Calcium Folinate, Fluorouracil, Irinotecan). Our findings revealed a significant correlation between EFTUD2 expression and key chemotherapy agents, such as 5-FU, capecitabine, and irinotecan (Supplementary Table 1). Additionally, an analysis of CTRP (Clinical Trials Reporting Program) database showed that EFTUD2 was significantly upregulated in the non-responsive group to several chemotherapy regimens, including FOLFOX and FOLFIRI (Fig. S1D). To elucidate the potential biological functions of EFTUD2 in CRC, we performed GSEA using the gene set linked to EFTUD2 from TCGA. Our results unveiled a statistically significant association between EFTUD2 and pathways related to the cell cycle, as well as drug metabolism cytochrome P450 pathways (Fig. S1E). Based on these findings, we posit that the overexpression of EFTUD2 may contribute to the chemoresistant phenotype in CRC, thereby exerting a substantial impact on patient prognosis.

### EFTUD2 expression was remarkably upregulated in CRC tissues

**Fig. 1:**
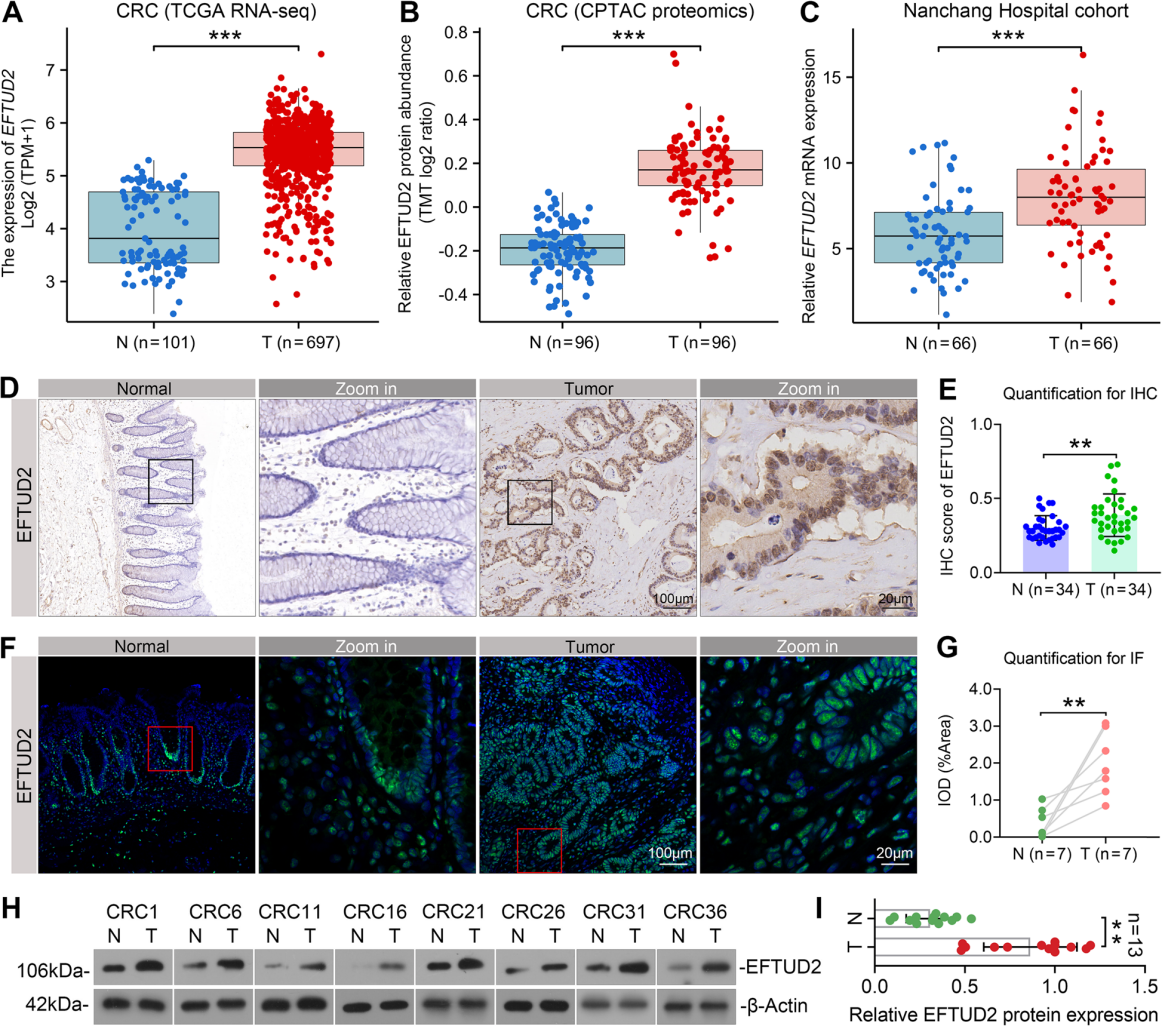
EFTUD2 is remarkably upregulated in CRC specimens. **A** Analysis of EFTUD2 mRNA expression in CRC tissues compared with adjacent normal tissues using TCGA database (Normal tissue, *n* = 101; Tumor tissue, *n* = 697). **B** Analysis of EFTUD2 protein expression in CRC tissues compared with paired adjacent normal tissues using CPTAC (Normal tissue, *n* = 96; Tumor tissue, *n* = 96). **C** RT-qPCR analysis of EFTUD2 mRNA expression in CRC tissues compared with adjacent normal tissues (Normal tissue, *n* = 66; Tumor tissue, *n* = 66). GAPDH is used as an endogenous control. **D** Representative images of EFTUD2 expression in 34 paired adjacent normal versus CRC tissue samples from the Nanchang Hospital cohort using IHC staining. **E** Quantification of EFTUD2 levels in CRC tissues and their corresponding adjacent normal tissues using IHC staining. **F** Representative images of EFTUD2 expression in CRC versus adjacent normal tissues using IF staining. **G** Quantification of EFTUD2 levels in CRC tissues and their corresponding adjacent normal tissues using IF staining. **H** Western blotting analysis of EFTUD2 protein expression in CRC tissues and their corresponding adjacent normal tissues. β-Actin is used as a loading control. **I** Quantification of the protein levels of EFTUD2 in CRC tissues and their corresponding adjacent normal tissues using Western blotting. Each bar represents the mean values ± SD. **P < 0.01; ***P < 0.001

To gain a comprehensive understanding of EFTUD2 expression patterns in CRC, we conducted a thorough analysis of its expression at both transcriptional and translational levels using data from the TCGA and CPTAC databases. Our results showed a significant upregulation of EFTUD2 expression in CRC tissues compared with adjacent normal tissues (Fig. 1A and B). To further validate these findings, we performed RT-qPCR on 66 postoperative clinical samples obtained from Nanchang Hospital. Consistently, the mRNA levels of EFTUD2 were significantly elevated in CRC tissues compared with their corresponding adjacent normal tissues (Fig. 1C). Additionally, IHC staining indicated significantly higher staining intensity and a greater proportion of EFTUD2-positive cells in paired CRC tissue samples compared with their corresponding adjacent normal tissues (Fig. 1D and E). Tissue IF staining and Western blotting experiments further corroborated these results (Fig. 1F-I). To summarize, our comprehensive analysis consistently demonstrate a substantial upregulation of EFTUD2 expression in CRC tissues compared with adjacent normal tissues.

#### EFTUD2 is an Independent prognostic indicator associated with poor outcomes in CRC patients

**Fig. 2:**
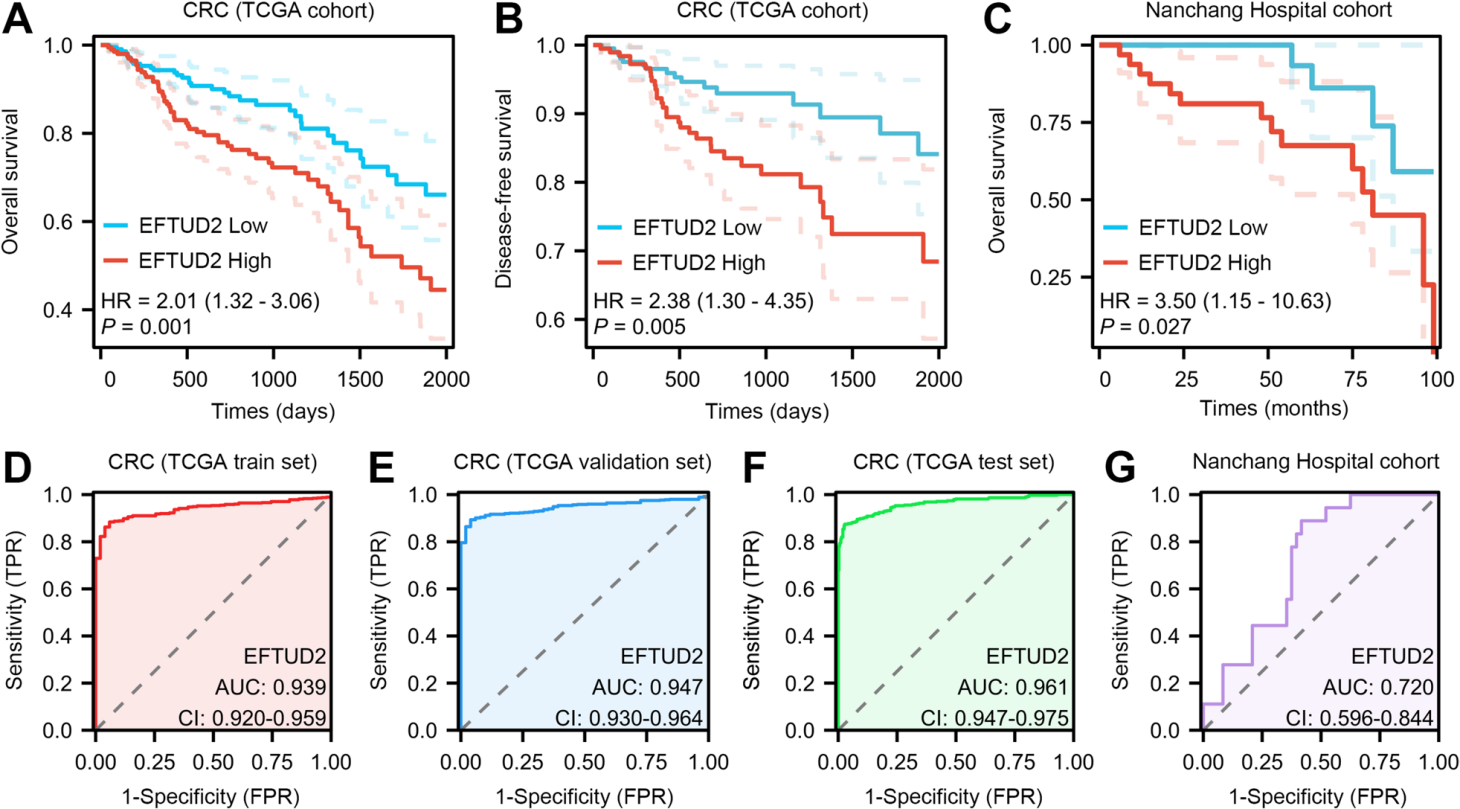
High EFTUD2 expression is a potential independent predictor of poor prognosis in CRC. **A** and **B** Kaplan-Meier analysis of overall survival (**A**) and disease-free survival (**B**) in two groups of CRC patients stratified by high and low expression of EFTUD2 using the data from the TCGA database. **C** Kaplan-Meier analysis of overall survival in CRC patients from the Nanchang Hospital cohort stratified by EFTUD2 expression (*n* = 66). In all analyses, the cutoff values for high and low expression are set at the ‘median’ of the EFTUD2 expression. **D-G** ROC curve analysis of the predictive accuracy and efficacy of EFTUD2 in CRC using the data from TCGA and the Nanchang Hospital cohort

In our quest to gain a comprehensive understanding of the implications of aberrant EFTUD2 expression in CRC, we embarked on an extensive analysis delving into the relationship between EFTUD2 expression and clinical-pathological parameters, leveraging data from patients within the TCGA cohort. Strikingly, our analysis results showed a strong correlation of EFTUD2 expression with several clinical parameters, including primary therapy outcome, TNM stage, and OS. Intriguingly, while these associations were significant, we did not observe a noteworthy link between EFTUD2 and lymphatic invasion, distant metastasis (pathologic M stage), and tumor location (anatomic neoplasm subdivision) (Supplementary Table 2). Subsequent Kaplan-Meier survival analysis on the clinical relevance of EFTUD2 in CRC, further revealing that patients with high expression levels of EFTUD2 had shorter OS and disease-free survival (DFS) compared with those with low EFTUD2 expression (OS: *P* = 0.001; DFS: *P* = 0.005, Fig. 2A and B). To validate, we performed Cox regression analysis using data from 66 CRC patients at Nanchang Hospital, which consistently indicated that the high-expression group exhibited significantly reduced OS in comparison to the low-expression group (*P* = 0.027, Fig. 2C). Moreover, univariate and multivariate Cox regression model analyses of a series of clinicopathological features for OS substantiated that an elevated EFTUD2 expression stands as a significant independent predictor of low OS in CRC patients (Table 1; Fig. S2A).

**Table 1:** Univariate and multivariate cox analysis of clinicopathological features for OS (TCGA cohort)

Characteristics	Total	Univariate analysis		Multivariate analysis	
		HR (95% CI)	*P*	HR (95% CI)	*P*
Gender	643		0.769		
Female	301	Reference			
Male	342	1.054 (0.744–1.491)	0.769		
Age	643		**< 0.001**		
<= 65	276	Reference		Reference	
> 65	367	1.939 (1.320–2.849)	**< 0.001**	1.363 (0.456–4.069)	0.579
Anatomic neoplasm subdivision	420		0.101		
Ascending Colon&Descending Colon	108	Reference			
Rectum&Sigmoid Colon&Transverse Colon	312	0.666 (0.414–1.071)	0.093		
Histological type	632		0.281		
Adenocarcinoma	549	Reference			
Mucinous adenocarcinoma	83	1.320 (0.810–2.151)	0.266		
Pathologic T stage	640		**0.001**		
T1&T2	131	Reference		Reference	
T3&T4	509	2.468 (1.327–4.589)	**0.004**	0.271 (0.057–1.282)	0.1
Pathologic N stage	639		**< 0.001**		
N0	367	Reference		Reference	
N1&N2	272	2.627 (1.831–3.769)	**< 0.001**	4.370 (0.891–21.446)	0.069
Pathologic M stage	563		**< 0.001**		
M0	474	Reference		Reference	
M1	89	3.989 (2.684–5.929)	**< 0.001**	1.827 (0.410–8.148)	0.43
Lymphatic invasion	581		**< 0.001**		
No	349	Reference		Reference	
Yes	232	2.144 (1.476–3.114)	**< 0.001**	2.197 (0.704–6.859)	0.175
CEA level	414		**< 0.001**		
<= 5	260	Reference		Reference	
> 5	154	2.620 (1.611–4.261)	**< 0.001**	1.035 (0.283–3.783)	0.958
Primary therapy outcome	312		**< 0.001**		
PD&SD	38	Reference		Reference	
PR&CR	274	0.109 (0.058–0.202)	**< 0.001**	0.089 (0.027–0.291)	**< 0.001**
EFTUD2	643		0.427		
Low	321	Reference		Reference	
High	322	1.101 (0.779–1.556)	**0.013**	1.464 (1.024–2.095)	**0.029**

To evaluate the predictive efficacy of EFTUD2 in CRC prognosis, we first analyzed TCGA database and conducted ROC curve analysis. Notably, our results showed remarkably high AUC values across three different cohorts: 0.939, 0.947, and 0.961 for the train set, validation set, and test set, respectively (Fig. 2D-F). Further, we conducted calibration curve analysis. The scale curves of 1-year, 2-year, and 3-year survival rates showed a remarkable degree of overlap between the actual survival rate and predicted survival rate (Fig. S2B), suggesting the robust predictive value of EFTUD2 in CRC prognosis. Moreover, our in-house cohort yielded a commendable AUC value of 0.720 (Fig. 2G). Collectively, these data demonstrate the robust predictive potency of EFTUD2 in determining the clinical outcome of CRC patients, establishing it a promising independent predictor of CRC prognosis.

### EFTUD2 impedes the sensitivity of CRC cells to 5-FU chemotherapy in vitro

**Fig. 3:**
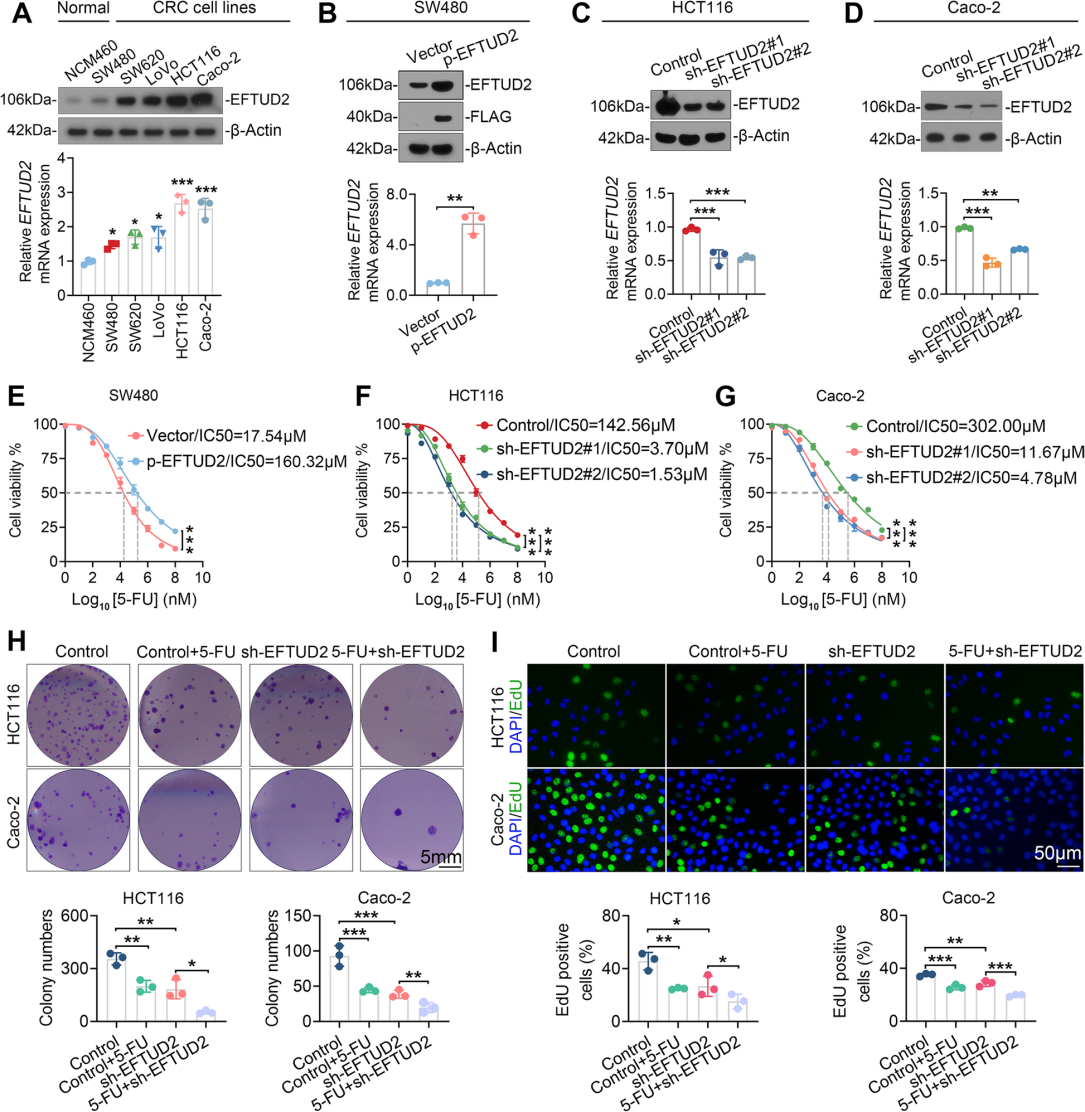
EFTUD2 attenuates the chemotherapy efficacy of 5-FU in vitro***.*** **A** Western blotting and RT-qPCR analyses of EFTUD2 expression in five CRC cell lines and normal colonic epithelial cells. β-Actin is used as a loading control in the Western blotting, and GAPDH serves as an endogenous control in RT-qPCR. **B-D** Western blotting and RT-qPCR analyses of changes in EFTUD2 expression levels in SW480, HCT116, and Caco-2 following overexpression or knockdown of EFTUD2. **E–G** MTT assay showing the effect of EFTUD2 overexpression or knockdown on cell viability in CRC cell lines exposed to a gradient of 5-FU concentrations. **H** and **I** Clone formation (**H**) and EdU (**I**) assays showing the effect of EFTUD2 on survival and proliferation potential in CRC cell lines exposed to 5-FU. Each bar represents the mean values ± SD. *P < 0.05; **P < 0.01; ***P < 0.001

To elucidate the implications of EFTUD2 in CRC’s chemosensitivity to 5-FU, we first assessed EFTUD2 expression across a spectrum of CRC cell lines. Our analysis unveiled an up-regulation of EFTUD2 in all CRC cell lines in comparison to the normal intestinal epithelial cell line NCM460 (Fig. 3A**)**. For experimental manipulation, we chose SW480, which displayed the lowest EFTUD2 expression among the cancer cell lines, for EFTUD2-overexpression experiments, while selecting Caco-2 and HCT116, both with higher EFTUD2 expression, for EFTUD2-knockdown experiments. The efficiency of these modifications was confirmed using Western blotting and RT-qPCR (Fig. 3B-D). Subsequently, we exposed CRC cells to increasing concentrations of 5-FU and determined their viability by calculating the corresponding IC50 values (Fig. S3A). Intriguingly, cell lines with overexpressed EFTUD2 exhibited a significant decline in sensitivity to 5-FU, suggesting a negative correlation between EFTUD2 expression and the chemosensitivity of CRC cells (Fig. 3E). Conversely, knocking down EFTUD2 expression increased the sensitivity of CRC cells to 5-FU (Fig. 3F and G). These findings were further corroborated through clone formation and EdU cell proliferation assays. Specifically, the knockdown of EFTUD2 expression reinforced the inhibitory effects of 5-FU on CRC cell survival proliferation (Fig. 3H and I), while EFTUD2 overexpression impaired these effects (Fig. S3B). Furthermore, the TUNEL assay revealed decreased apoptosis in EFTUD2-overexpressing cells (Fig. S3C), while EFTUD2 knockdown enhanced the apoptotic process (Fig. S3D) when exposed to 5-FU. Taken together, our data suggest that reducing EFTUD2 expression effectively improves the sensitivity of CRC cells to 5-FU chemotherapy.

### EFTUD2 hampers the sensitivity of CRC cells to 5-FU chemotherapy in vivo

**Fig. 4:**
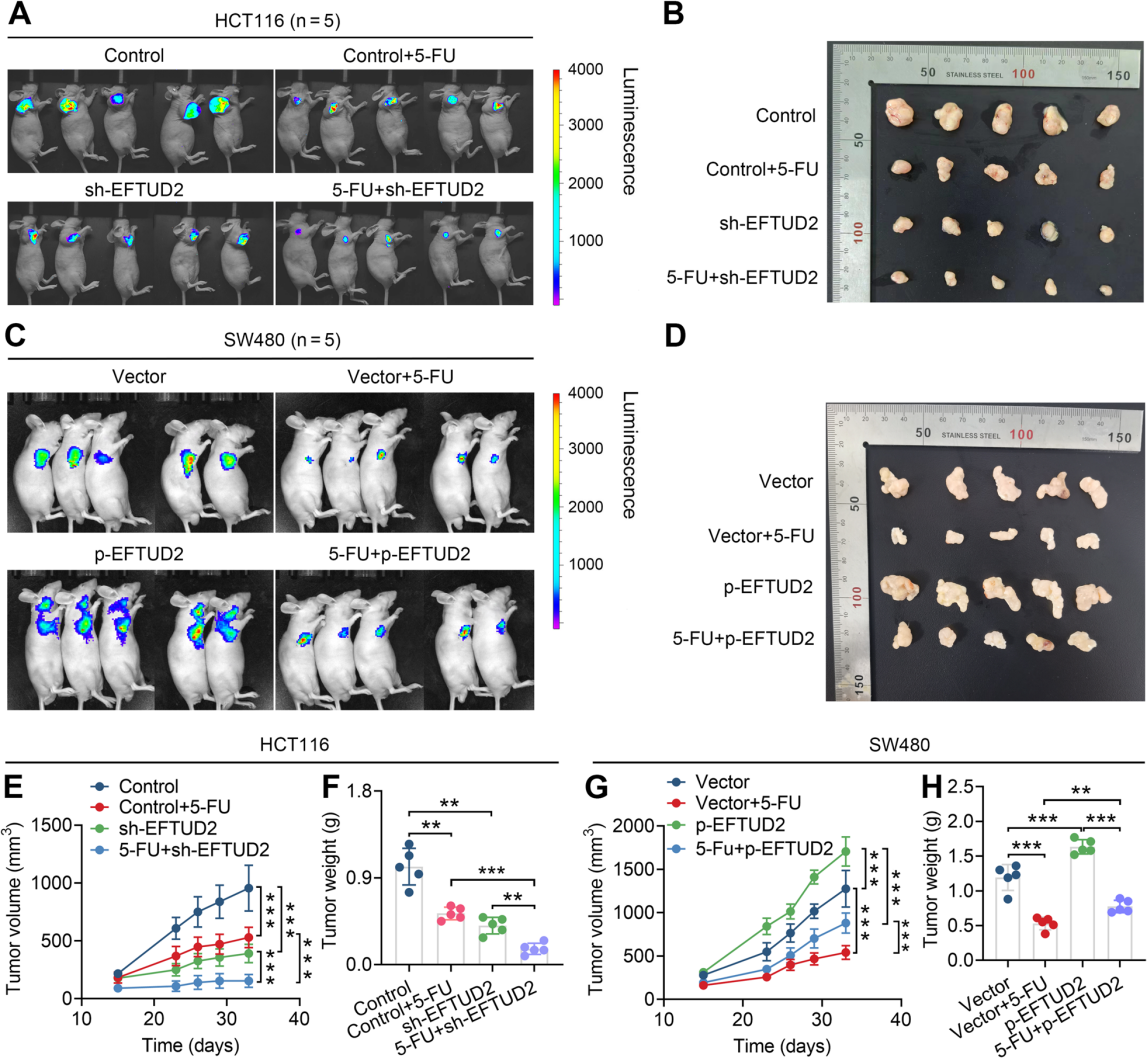
EFTUD2 hampers the chemotherapy efficacy of 5-FU in vivo. **A** and **B** HCT116 cells transfected with sh-EFTUD2 are subcutaneously injected into BALB/c nude mice, and tumor volumes are assessed using an animal live imaging system (IVIS) (**A**). Representative images of tumors harvested from different groups of mice (*n* = 5) are displayed (**B**). **C** and **D** Similarly, SW480 cells transfected with p-EFTUD2 are subcutaneously injected (**C**), and representative images of tumors from these mice (*n* = 5) are presented (**D**). **E** and **G** Growth curves illustrating the changes in tumor volume in mice across various groups (volume = 0.52 × length × width^2^) (*n* = 5). **F **and **H** Differences in tumor weight between the groups (*n* = 5). Each bar represents the mean values ± SD, **P < 0.01, ***P < 0.001

We sought to confirm the role of EFTUD2 in regulating the chemosensitivity of 5-FU in vivo using a CRC/CDX model. To achieve this, we tagged HCT116 and SW480 cells with luciferase fluorescent markers and transplanted them into BALB/c nude mice, creating different experimental groups (i.e. Control, 5-FU + Control, sh-EFTUD2, 5-FU + sh-EFTUD2; *n* = 5 for each group). Following 5-FU treatment, we monitored luciferase expression levels by IVIS at specified time points throughout the modeling period. Changes in fluorescence intensity served as an indicator of tumor cell proliferation. Consistent with our in vitro cellular assays, the results of in vivo imaging showed a significant reduction in tumor mass and volume upon EFTUD2 knockdown, specifically in the sh-EFTUD2/HCT116 group versus the control/HCT116 group. Notably, inhibiting EFTUD2 expression significantly synergistically increased the sensitivity of HCT116 cells to 5-FU chemotherapy, as observed in the 5-FU + sh-EFTUD2 group versus 5-FU + Control group (Fig. 4A, B, E, F). Conversely, the overexpression of EFTUD2 in SW480 yielded contrasting outcomes (Fig. 4C, D, G, H). Taken together, the results of in vivo experiments using the CRC/CDX model further support our hypothesis that inhibiting EFTUD2 expression synergistically enhances the chemosensitivity of CRC cells to 5-FU.

### EFTUD2 interacts with c-MYC protein in CRC cells

**Fig. 5:**
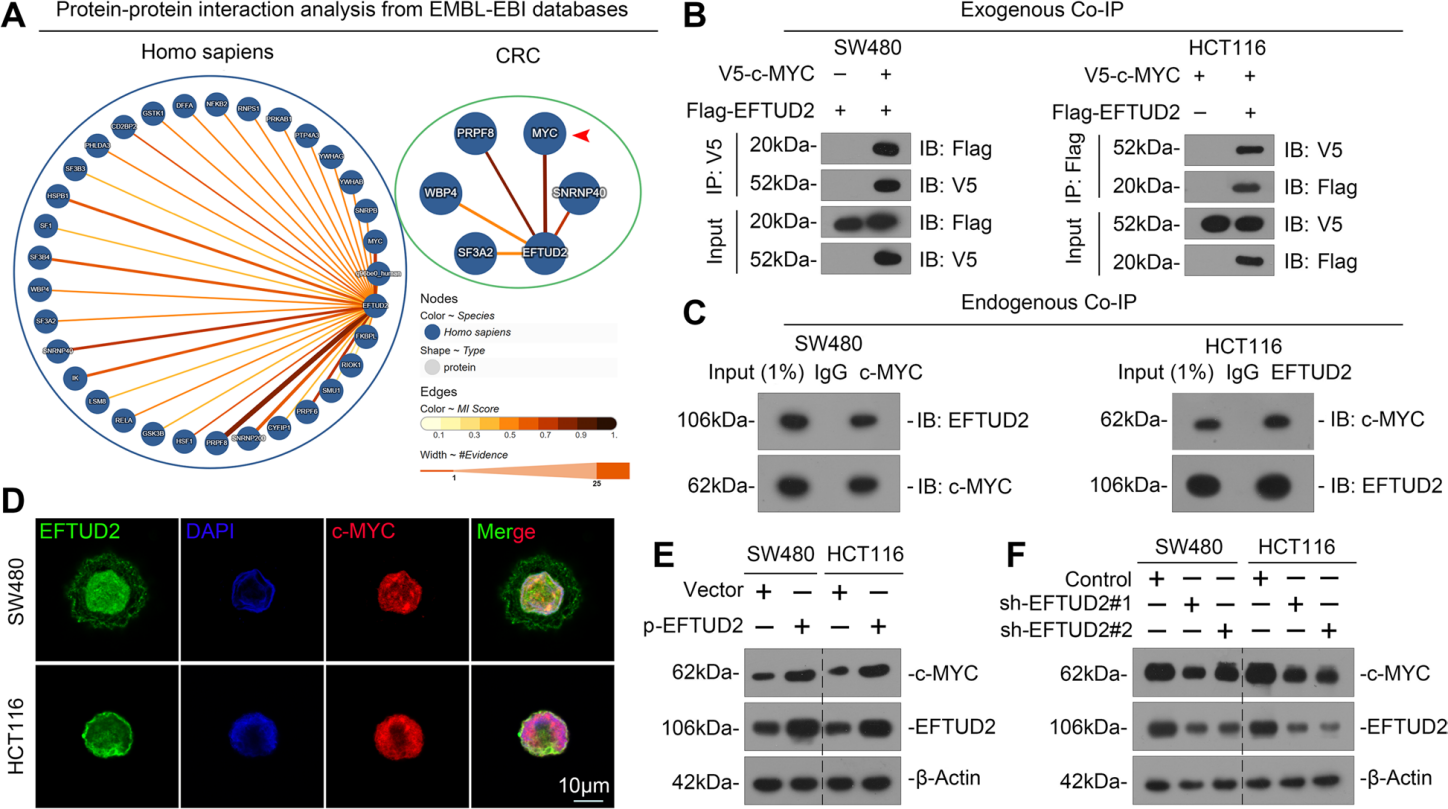
EFTUD2 interacts with c-MYC and increases c-MYC protein abundance in CRC. **A** Protein-protein interaction analysis between EFTUD2 and c-MYC protein using EMBL-EBI database. **B** and **C** Co-IP and Western blotting analyses detecting the interaction between EFTUD2 and c-MYC by modulating their exogenous (**B**) and endogenous (**C**) expression in SW480 and HCT116 cells. **D** IF staining showing co-localization of EFTUD2 and c-MYC in SW480 and HCT116 cells. DAPI (blue), EFTUD2 (green), and c-MYC (red). **E** and **F** Western blotting analysis of the impact of overexpression or knockdown of EFTUD2 on the expression of c-MYC in SW480 and HCT116 cells

To elucidate the potential mechanisms underlying the role of EFTUD2 in enhancing cellular sensitivity to chemotherapy in CRC, we initiated an investigation on the protein interaction network involving EFTUD2 using EMBL-EBI database. Affinity mass spectrometry data revealed a robust interaction between EFTUD2 and c-MYC protein (Fig. 5A). In addition, a co-expression heat map derived from the TCGA database showed a significantly positive correlation between the expressions of EFTUD2 and MYC (Fig. S4A). MYC expression was markedly upregulated in CRC tissues, implying a potentially pivotal role of EFTUD2 in the context of MYC (Fig. S4B-F).

To substantiate this correlation, we investigated whether EFTUD2 and c-MYC protein could indeed interact with each other. Co-IP results showed interactions between EFTUD2 and c-MYC protein, both in exogenous and endogenous contexts (Fig. 5B and C). Further, confocal microscopy confirmed the co-localization of EFTUD2 and c-MYC in CRC cells (Fig. 5D). To gain a deeper understanding of the functional implications of their physical binding, we manipulated EFTUD2 expression in CRC cells through overexpression and knockdown experiments. Subsequent Western blotting analysis showed that the upregulation of EFTUD2 significantly promoted c-MYC protein expression, while the inhibition of EFTUD2 remarkably reduced c-MYC expression (Fig. 5E and F). These findings suggest that EFTUD2 plays a role in safeguarding the protein abundance of c-MYC.

### EFTUD2 stabilizes the protein expression of c-MYC by inhibiting its ubiquitin proteasome system (UPS)-mediated degradation in CRC cells

**Fig. 6:**
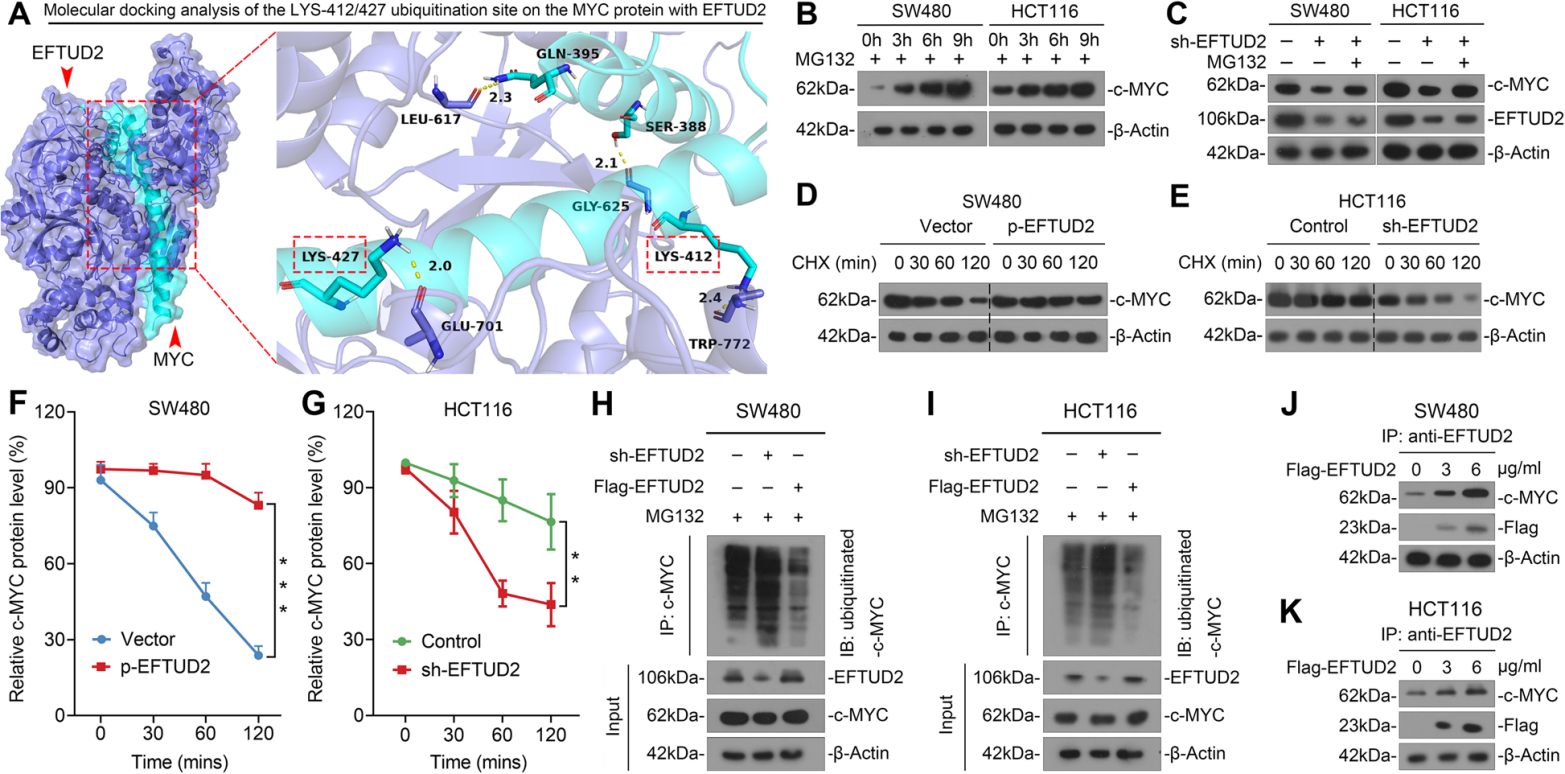
EFTUD2 stabilizes c-MYC protein by inhibiting UPS-mediated degradation in CRC cells. **A** The top-ranked 3D docking conformation of EFTUD2 and c-MYC is shown using HDOCK. **B** Western blotting analysis detecting protein levels of c-MYC in SW480 and HCT116 treated with MG132 (10 µM) at different time intervals (0, 3, 6, 9 h) **C** Western blotting analysis showing changes in c-MYC expression before and after knockdown of EFTUD2 in SW480 and HCT116 simultaneously treated with MG132 (10 µM) for 6 h. **D-G** Protein half-life assay (**D** and **E**) and subsequent quantification (**F** and **G**) showing changes in c-MYC protein half-life upon modulation of EFTUD2 expression in SW480 and HCT116 treated with 20 µg/mL of CHX and transfected with sh-EFTUD2 or p-EFTUD2 plasmids. Protein is extracted at the indicated time points (0, 30, 60, 120 min). **H** and **I** Co-IP analysis detecting changes in the level of ubiquitination bound to c-MYC protein upon modulation of EFTUD2 expression in SW480 and HCT116 cells treated with MG132 (10 µM) and transfected with sh-EFTUD2 or Flag-EFTUD2 plasmids. **J** and **K** Co-IP analysis detecting changes in the level of c-MYC in SW480 and HCT116 cells transfected with a gradient of Flag-EFTUD2 plasmids. Each bar represents the mean values ± SD, **P < 0.01, ***P < 0.001

To establish if EFTUD2 plays a role in the post-translational regulation of c-MYC, we initiated a molecular docking analysis of protein three-dimensional structures, unveiling a stable interaction between EFTUD2 and c-MYC at the ubiquitination sites LYS-412/427 of c-MYC (Fig. 6A). Notably, c-MYC is known to have a short half-life of 30 min, making it subject to rapid degradation through UPS [32, 33]. To ascertain the involvement of the UPS in c-MYC protein degradation, we employed MG132, a widely used proteasome inhibitor, leading to a gradual accumulation of c-MYC protein over time (0 to 9 h) (Fig. 6B). Intriguingly, knocking down EFTUD2 resulted in rapid degradation of c-MYC; however, MG132 treatment effectively reversed this effect, suggesting a pivotal role of EFTUD2 in the UPS-dependent degradation of c-MYC protein in CRC cells (Fig. 6C). To elucidate the mechanisms underlying c-MYC stability and turnover, we conducted a protein half-life assay using cycloheximide (CHX). Overexpressing EFTUD2 in CRC cells substantially preserved the abundance of c-MYC protein in the presence of CHX (Fig. 6D and F), whilst knocking down EFTUD2 accelerated c-MYC degradation (Fig. 6E and G). These findings suggest the significant role of EFTUD2 in controlling the half-life and stability of c-MYC.

To further confirm EFTUD2’s role in stabilizing c-MYC by impeding UPS-mediated degradation, we conducted a ubiquitination assay. Co-IP results showed that downregulation of EFTUD2 expression led to an increase in c-MYC ubiquitination levels, while upregulation of EFTUD2 expression significantly decreased ubiquitination (Fig. 6H and I). Finally, we sought to corroborate the significance of the physical interaction of EFTUD2 and c-MYC in controlling c-MYC stability. Co-IP results revealed that an increasing EFTUD2-c-MYC complex resulted in a steady increase of c-MYC levels in CRC cells (Fig. 6J and K). Collectively, these findings demonstrate that EFTUD2 contributes to the stabilization of c-MYC expression by preventing its ubiquitination, ultimately influencing the malignant biological phenotype of CRC cells, including their sensitivity to the 5-FU treatment.

### c-MYC promotes EFTUD2 transcriptional expression

**Fig. 7:**
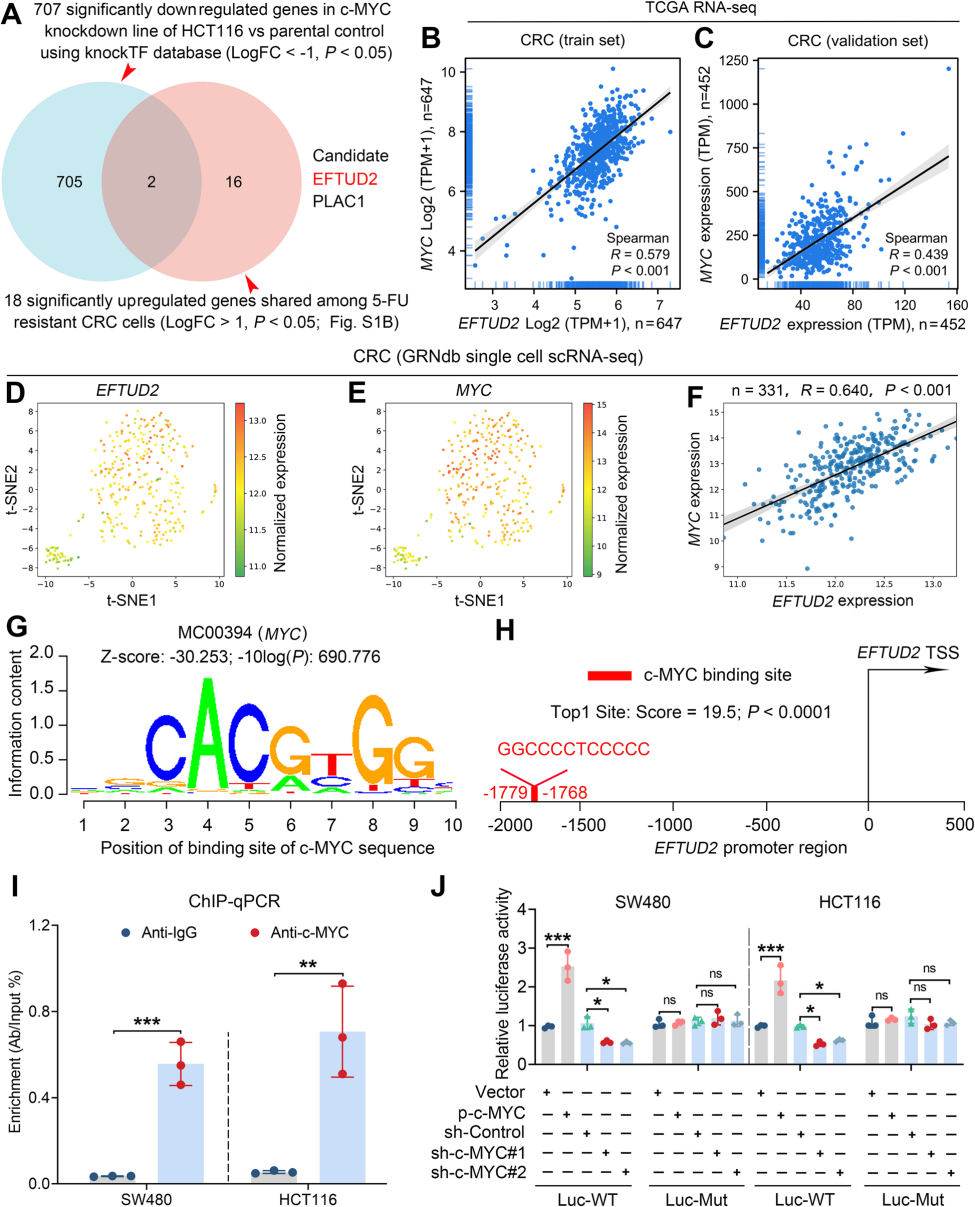
c-MYC promotes the transcriptional expression of EFTUD2. **A** Venn diagram displays the intersection of target genes downregulated by c-MYC knockdown in HCT116 cells with those upregulated, as shown in Fig. S1B. **B** and **C** Scatterplots illustrate the correlation between EFTUD2 and c-MYC in the TCGA cohort. **D-F** Feature plots (**D** and** E**) and a scatterplot (**F**) depict the correlation between EFTUD2 and c-MYC in the TCGA-CRC single cell transcriptome. **G** Analysis using Cistrome Data Browser and JASPAR database show the predicted c-MYC binding motifs in the EFTUD2 promoter. **H** Schematic illustrates the top predicted c-MYC binding site within the EFTUD2 promoter region. **I** ChIP-qPCR analysis show c-MYC directly binding to the specific site on the EFTUD2 promoter in SW480 and HCT116 cells. **J** Dual luciferase reporter assay shows luciferase activity controlled by EFTUD2 wild-type (Luc-WT) but not the mutant (Luc-Mut) promoter in a c-MYC dependent manner. Each bar represents the mean values ± SD, *P < 0.05; **P < 0.01; ***P < 0.001

The c-MYC transcriptional factor is a well-known oncogenic master regulator [34]. In our investigation of potential target genes of c-MYC in the context of CRC chemoresistance, we employed multiple analyses. Firstly, leveraging the knockTF database, we identified 707 genes that were significantly downregulated genes in a c-MYC knockdown model of HCT116 versus parental control cells [24]. Subsequently, we intersected these genes with a set of 18 significantly upregulated genes shared among 5-FU resistant CRC cells (Fig. S1B). Surprisingly, this approach revealed EFTUD2 as a putative transcriptional target of c-MYC (Fig. 7A). To further explore the connection between c-MYC and EFTUD2, we conducted GSEA using TCGA database. Our analysis suggested a notable enrichment of MYC/MAX protein complex, which is known to promote the transcription of EFTUD2 (Fig. S5A). Furthermore, we analyzed the expression correlation between EFTUD2 and c-MYC using RNA-seq data derived from TCGA-CRC projects. The resulting expression correlation scatter plot showed a significant positive correlation between EFTUD2 and c-MYC in both the training and validation datasets (Fig. 7B and C). Additionally, a single-cell RNA sequencing analysis using GRNdb database confirmed that EFTUD2 expression was significantly correlated with c-MYC expression at the transcriptional level (Fig. 7D-F).

To gain an understanding of potential transcription factor (TF) binding sites within EFTUD2 promoter region, we conducted JASPAR analysis, revealing the presence of c-MYC binding motifs within the EFTUD2 promoter region in CRC cell lines (Fig. 7G). Notably, the site located at -1779 to -1768 (GGCCCCTCCCCC) upstream of the EFTUD2 transcription start site was the top-ranked predicted binding sites (Fig. 7H). To validate the presence of this binding site, we conducted a ChIP assay followed by RT-qPCR analysis, showing that c-MYC directly bound to EFTUD2 promoter (Fig. 7I). Further, functional validation was performed through a dual luciferase reporter assay. The assay showed an increase in luciferase activity when the EFTUD2 promoter was introduced along with c-MYC. Conversely, downregulation of c-MYC significantly attenuated the luciferase activity in both SW480 and HCT116 cell lines. Importantly, this effect was abolished when the binding site within the EFTUD2 promoter was mutated (Fig. 7J). Collectively, our data demonstrate that c-MYC directly binds to the EFTUD2 promoter and promotes its transcription, suggesting the existence of a positive feedback loop between c-MYC and EFTUD2.

### c-MYC-dependent EFTUD2/c-MYC positive feedback loop impedes the chemotherapy sensitivity of CRC

**Fig. 8:**
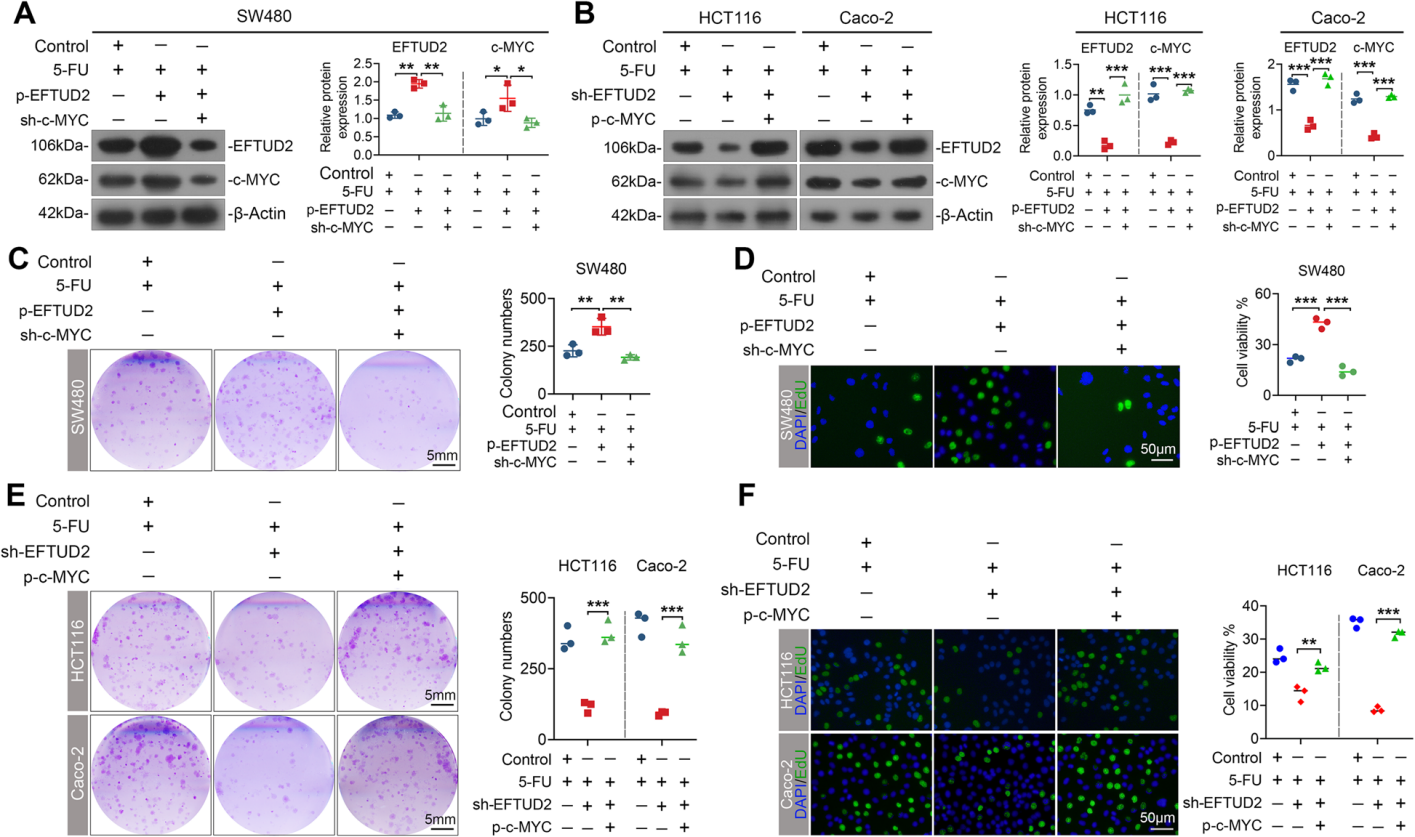
The effect of EFTUD2 on attenuating the chemotherapy efficacy of 5-FU is dependent on c-MYC stabilization. **A** and **B** Western blotting analysis of changes in EFTUD2 protein levels before and after modulation of c-MYC expression in SW480 (**A**), HCT116, and Caco-2 (**B**) cells treated with 5-FU. **C** and **D** Clone formation (**C**) and EdU (**D**) assays showing the influence of EFTUD2 upregulation on cell survival and proliferation before and after c-MYC knockdown in SW480 cells treated with 5-FU. **E** and **F** Clone formation (**E**) and EdU (**F**) assays showing the influence of EFTUD2 downregulation on cell survival and proliferation before and after c-MYC overexpression in HCT116 and Caco-2 cells treated with 5-FU. Each bar represents the mean values ± SD, *P < 0.05; **P < 0.01; ***P < 0.001

To determine the significance of c-MYC within the EFTUD2/c-MYC loop in diminishing chemotherapy sensitivity in CRC, we modulated the expression of c-MYC in CRC cells exposed to 5-FU. As expected, downregulating c-MYC resulted in a significant decrease in EFTUD2 protein expression (Fig. 8A), whilst upregulating c-MYC led to an increase in EFTUD2 levels (Fig. 8B). Notably, the clone formation and EdU assays showed that EFTUD2 overexpression significantly enhanced cell survival and proliferation under 5-FU treatment; however, such effects on impairing the chemotherapy sensitivity of CRC cell to 5-FU was abolished upon down-regulation of c-MYC (Fig. 8C and D). In keeping, c-MYC overexpression effectively reversed the enhanced chemosensitivity induced by EFTUD2 knockdown (Fig. 8E and F). In conclusion, our results suggest a c-MYC-dependent EFTUD2/c-MYC positive feedback loop, impacting chemotherapy sensitivity of CRC cells.

## Discussion

Accumulating studies have showed that mechanisms underlying chemotherapy resistance primarily involve alterations in cell proliferation and apoptosis [35] where transcriptional regulation and post-translational modifications assume pivotal roles [36, 37]. Our study aims to elucidate the specific function of EFTUD2 and its underlying mechanism in modulating the chemotherapy sensitivity of CRC cells. Here, we identified an upregulation of EFTUD2 in 5-FU-resistant CRC cells. Notably, EFTUD2 also served as an independent prognostic indicator associated with poor outcomes in CRC patients. Furthermore, EFTUD2 overexpression impairs the sensitivity of CRC cells to 5-FU chemotherapy, while EFTUD2 knockdown enhances this sensitivity, both in vitro and in vivo*.* Mechanistically, we demonstrated that EFTUD2 stabilized the protein expression of c-MYC by inhibiting its ubiquitin proteasome system (UPS)-mediated degradation. In turn, c-MYC, a known oncogenic master regulator, promotes EFTUD2 transcriptional expression, effectively forming a positive feedback loop and thereby impacting chemotherapy sensitivity of CRC cells. Therefore, our results unveil a novel mechanism by which EFTUD2/c-MYC positive feedback loop impedes the chemotherapy sensitivity of CRC.

An array of studies revealed the canonical role of EFTUD2 within the spliceosome complex, a critical machinery responsible for pre-mRNAs splicing and the generation of mature mRNAs [8]. Hence, we asked whether EFTUD2 could affect the sensitivity of CRC cells to 5-FU chemotherapy by modulating the maturation of a class of mRNAs or a specific mRNA. To address this, we sought to identify the mRNA(s) that could possibly be regulated by EFTUD2 via its canonical role in mRNA maturation, particularly in the context of CRC chemoresistance. Revisiting the primary focus of our study, we had set out to discover novel regulators involved in CRC chemoresistance and consequently conducted a comprehensive transcriptional analysis by comparing parental and 5-FU resistant cells across three GEO datasets (GSE166900, GSE81005, GSE81008). This analysis unveiled 18 significantly upregulated genes, including EFTUD2 as a prominent candidate, shared among CRC chemoresistant cells (Fig. S1A and B). Thus, it was logical to establish if EFTUD2 could affect chemoresistance through its canonical function by influencing these 17 other genes. We hypothesized that elevated levels of EFTUD2 may result in an upregulation of these 17 genes by promoting their mRNA maturation. To explore this, we conducted a Spearman analysis to assess the correlation between EFTUD2 and the 17 genes, focusing on the transcript levels within 5-FU-resistant cell lines (Fig. S6A). This comprehensive analysis revealed relatively weak correlation between EFTUD2 and the 17 genes. Further, we expanded our approach and still considered the three genes: SLC2A1 (R = 0.305), L1CAM (R = 0.203), and IL32 (R = 0.200), which exhibited commendably positive correlations with EFTUD2, as illustrated in the heat map generated using the ggplot2 package. Subsequently, we extended our investigation to evaluate the expression of these genes and their impact on the overall survival (OS) of colorectal cancer patients (Fig. S6 B-D). Unfortunately, our analysis revealed that SLC2A1, L1CAM, and IL32 lacked significant prognostic relevance in the context of CRC. Taken together, we postulate that it is less likely that EFTUD2 plays a pivotal role in driving the malignant phenotypes of CRC, including chemoresistance, through its canonical RNA spliceosomal function.

Chemoresistance is a complex phenomenon in which cancer cells develop resistance to the cytotoxic effects of chemotherapy drugs, presenting a formidable challenge in the field of cancer treatment. Ubiquitination, a tightly regulated post-translational modification process, plays a crucial role in the degradation of key proteins, particularly those involved in cell cycle [38] regulation and DNA repair [39]. Consequently, dysregulation of the ubiquitin-proteasome system can result in the overexpression of anti-apoptotic proteins and drug efflux transporters [40], ultimately contributing to the development of chemoresistance. In our study, we have unveiled a previously unrecognized mechanism through which EFTUD2 protects the protein abundance of c-MYC by inhibiting its degradation through the ubiquitin-proteasome system (UPS). Notably, Ge et al. [41] reported that the deubiquitinase USP16 promotes the proliferation of castration-resistant prostate cancer cells by deubiquitinating and stabilizing c-MYC. Additionally, Xia et al. [42] showed that methyltransferase 5, N6-adenosine (METTL5) can also stabilize c-MYC through several pro-oncogenic USP5-c-MYC signaling cascades, thereby enhancing the proliferation and migration of hepatocellular carcinoma (HCC) cells. Zhu et al. [43] demonstrated that the long non-coding RNA LINC00942 promotes chemoresistance in gastric cancer by inhibiting the degradation of the potential N6-methyladenosine (m^6^A) recognition protein MSI2, which, in turn, stabilizes *c-Myc* mRNA with m^6^A modifications. Intriguingly, Zhou et al. [44] suggest that EFTUD2 may have a non-canonical role as a ubiquitin modification enzyme in the degradation of YTH domain family protein 3 (YTHDF3) in HCC, indicating the need for further investigation into its diverse functions. Collectively, our results shed new light on the ubiquitination of c-MYC, presenting a promising avenue for the development of innovative therapeutic strategies aimed at overcoming chemoresistance in colorectal cancer.

In our study, our primary objective was to investigate the pivotal role of EFTUD2 in CRC cell proliferation and its influence on sensitivity to 5-FU chemotherapy. Our research began by identifying a significant upregulation of EFTUD2 in 5-FU-resistant colorectal cancer tissues, and subsequent Gene Set Enrichment Analysis (GSEA) indicated its association with chemotherapy-resistant phenotypes (Fig. S1). Thus, our primary focus was to unravel the mechanisms underlying how EFTUD2 regulates chemoresistance in CRC cells. In the meantime, we recognize that at the tissue and clinical levels, the development of cellular chemotherapy resistance can lead to tumor recurrence, metastasis, and an elevated risk of complications [45]. Furthermore, at the molecular and cellular levels, chemoresistance is intricately linked to various malignant phenotypes, including an increased proportion of stem cells, epithelial-mesenchymal transition (EMT) [46], dysregulation of DNA replication [47], and abnormalities in metabolic and epigenetic reprogramming [48]. Recurrent metastasis represents one facet of this intricate interplay.

While the direct examination of CRC cell metastasis was not within the scope of our study, we acknowledge the pivotal role of metastasis in chemotherapy tolerance and appreciate the need for further investigation into its connection with EFTUD2. To address this, we explored the metastatic potential of the EFTUD2/c-MYC axis, with a particular focus on EMT as an indicator. We assessed changes in the expression of EMT markers following EFTUD2 overexpression using RT-qPCR. Our results unveiled a significant increase in the expression levels of *Vimentin*, *ZEB1*, and *Slug*, accompanied by a significant decrease in *E-cadherin* expression (Fig. S7). These findings suggest that EFTUD2 may promote the EMT phenotype, potentially contributing to tumor metastasis. Undoubtedly, this area warrants in-depth exploration in future research.

c-*MYC* is a well-known oncogene that regulates many cellular processes, including cell cycle, metabolism, and apoptosis [15]. c-MYC is also frequently overexpressed or amplified in CRC and confers chemoresistance by modulating drug transporters, DNA repair, and anti-apoptotic pathways [49]. Our study underscores the importance of the positive feedback loop of the EFTUD2/c-MYC axis, contributing to the attenuation of chemotherapy sensitivity. While our analysis of ChIP-seq data from the Cistrome Data Browser indicated a pronounced enrichment of c-MYC binding peaks located away from the EFTUD2 promoter region in multiple CRC cells (Fig. S2B), our ChIP-qPCR and dual luciferase reporter assays provided direct evidence of c-MYC’s functional binding to the EFTUD2 promoter. These findings lay the foundation for future research, suggesting that the downstream execution may predominantly hinge on c-MYC as a master transcription factor. At the molecular level, c-MYC may precisely modulate CRC chemotherapy sensitivity through known or novel downstream mechanisms. For instance, high c-MYC expression in colon cancer is strongly associated with tumor recurrence in patients undergoing adjuvant chemotherapy with 5-fluorouracil. This association is attributed to the activation of the ABC family transporter ABCB5 by c-MYC, leading to increased drug efflux [50]. Moreover, overexpression of c-MYC results in the expansion of intestinal stem and progenitor cells, which in turn increases organoid formation and proliferation. These mechanisms might be regulated by the PCNA-associated factor/Wnt/β-catenin signaling, Hedgehog signaling, cell migration-inducing protein (CEMIP)-dependent pathway through ERK1/2, or silencing of the PR/SET Domain1 (PRDM)1 gene [51]. Further, solute carrier proteins (SLCs) play a significant role in maintaining cellular metabolic homeostasis via the export and import of drugs, contributing to chemoresistance. SLC1A5, known as Alanine-Serine-Cysteine Transporter 2 (ASCT2), stands as a predominant member of SLCs and is a major target of the oncogene c-*MYC*. Therefore, c-MYC may be involved in chemoresistance by regulating the expression of SLCs [52].

## Conclusions

**Fig. 9:**
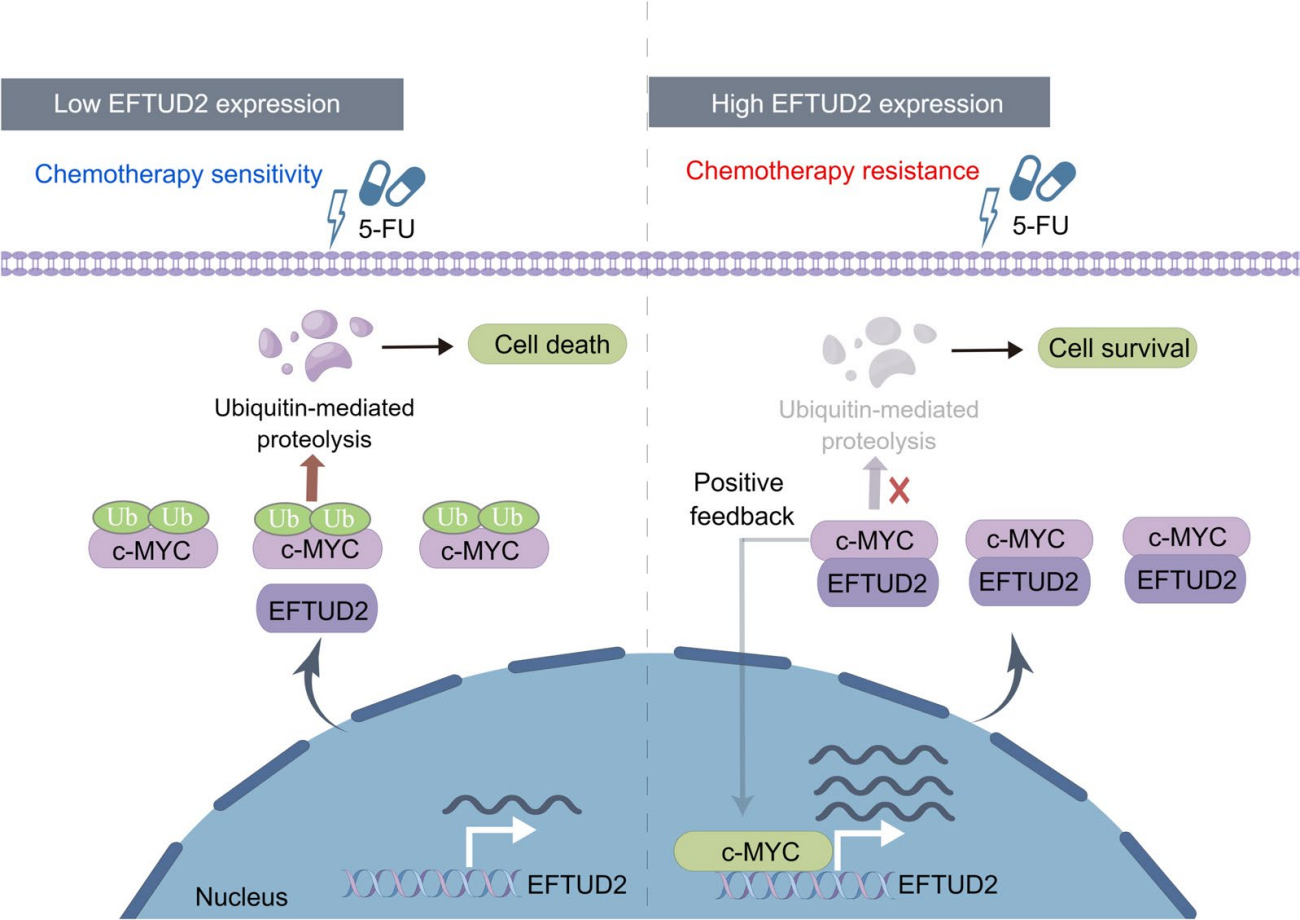
Diagram of the regulatory mechanism through which EFTUD2 attenuates the chemotherapy efficacy of 5-FU by preventing UPS-mediated degradation of c-MYC in CRC

In this study, we have identified that elevated expression of EFTUD2 represents a potential independent prognostic indicator and is associated with adverse clinical outcomes in CRC patients. Notably, the knockdown of EFTUD2 has been shown to significantly enhance the chemosensitivity of CRC cells to 5-FU both in vitro and in vivo. Mechanistically, EFTUD2 stabilizes the expression of c-MYC by preventing the binding of ubiquitin to c-MYC, thereby inhibiting UPS-mediated degradation of c-MYC. This establishment of a positive feedback loop results in an upregulation of EFTUD2 transcription (Fig. 9). Consequently, further investigations into the underlying mechanism and biological function of the EFTUD2/c-MYC axis in cancer development are anticipated. Via focusing on either the loop or EFTUD2 per se, such studies hold promise in addressing the challenge of c-MYC as a formidable drug target.

### Supplementary Information

**Additional file 1: Fig. S1. **EFTUD2 is significantly upregulated in 5-FU chemotherapy-resistant cells of CRC, related to Fig. 1. **A** Analysis of the differential expression genes (DEGs) expression in 5-FU resistant CRC cell lines compared with parental cell lines using three GEO datasets (GSE166900, GSE81005, GSE81008). **B** Analysis of 18 significantly upregulated genes shared among CRC chemoresistant cells using Venn diagram. **C** Multivariate Cox regression analysis of 18 candidate genes and identification of top four genes (*EFTUD2*, *VIM*, *EFNB2*, and *PLAC1*) using TCGA. **D** Analysis of EFTUD2 expression in the non-responsive group compared with response group to FOLFOX and FOLFIRI using CTRP. **E **GSEA using the gene set linked to EFTUD2 in CRC from TCGA. **P < 0.01. **Fig. S2. **High EFTUD2 expression is a potential independent predictor of poor prognosis in CRC, related to Fig. 2. **A** Analysis of EFTUD2 in predicting survival probability in CRC patients based on Cox regression analysis using Nomogram-related model. **B** Calibration curve analysis depicting the differentiation between predicted and actual survival rates of the model at different time points (1-year, 2-year, and 3-year). **Fig. S3. **EFTUD2 attenuates the chemotherapy efficacy of 5-FU in vitro, related to Fig. 3. **A** MTT assay showing the influence of various concentrations of 5-FU on viability of SW480, HCT116, and Caco-2 cells. B Clone formation and EdU assays showing the influence of EFTUD2 upregulation on cell survival and proliferation in SW480 cells treated with 5-FU. **C** and **D** TUNEL apoptosis assays showing the influence of EFTUD2 modulation on apoptosis in SW480, HCT116, and Caco-2 cells treated with 5-FU. Each bar represents the mean values ± SD, *P < 0.05; **P < 0.01; ***P < 0.001. **Fig. S4. **Correlation and expression analysis of EFTUD2 with candidate genes, related to Fig. 5. **A** Heat map showing the top five genes correlated with EFTUD2 in CRC using TCGA. **B-F** Differentiation expression analysis showing the mRNA expression levels of *MYC*, *PRPF8*, *WBP4*, *SF3A2*, and *SNRNP40* in CRC tissues compared with adjacent normal tissues using TCGA. ***P < 0.001. **Fig. S5. **MYC promotes the transcription of EFTUD2, related to Fig. 7. **A** GSEA showing the transcription factors related to EFTUD2 in CRC using TCGA. **B** ChIP-seq analysis of MYC binding peaks on EFTUD2 using Cistrome Data Browser. **Fig. S6. **Correlation and overall survival analysis of EFTUD2 with 17 significantly upregulated genes shared in CRC chemoresistant cells. **A** Heat map showing the correlation between EFTUD2 and 17 significantly upregulated genes using the TCGA. **B **Kaplan-Meier analysis of overall survival in two groups of CRC patients stratified by high and low expression of top three genes (*SLC2A1*, *L1CAM*, and *IL32*) using TCGA. **Fig. S7.** Overexpressing EFTUD2 promotes the epithelial-mesenchymal transition (EMT) phenotype. **A** and **B** Analysis of EMT markers (*Vimentin*, *E-cadherin*, *ZEB1*, and *Slug*) after overexpressing EFTUD2 in SW480 and HCT116 cells using RT-qPCR. Each bar represents the mean values ± SD, *P < 0.05; **P < 0.01; ****P < 0.0001.** Supplementary Table 1. **Correlation analyses of EFTUD2 with sensitivity to common chemotherapeutic drugs in CRC. **Supplementary Table 2.** Correlation analyses between EFTUD2 expression and clinicopathological characteristics of CRC patients using TCGA. **Supplementary Table 3.** The sequence of primers used in Fig.7K and Fig. 8

## Data Availability

All data from this study are available upon request from the corresponding author.

## Abbreviations

CRC: Colorectal cancer
EFTUD2: Elongation factor Tu GTP binding domain containing 2
5-FU: 5-fluorouracil
ChIP: Chromatin immunoprecipitation
Co-IP: Co-immunoprecipitation
CDX: Cell-derived xenograft
IF: Immunofluorescence
IHC: Immunohistochemistry
CAC: Colitis-associated cancer
pre-mRNAs: Precursor mRNAs
COAD: Colon adenocarcinoma
READ: Rectum adenocarcinoma
ROC: Receiver operating characteristic
GSEA: Gene set enrichment analysis
OS: Overall survival
DFS: Disease-free survival
UPS: Ubiquitin-proteasome system
