# Supplementary material for: The feedback loop of EFTUD2/c-MYC impedes chemotherapeutic efficacy by enhancing EFTUD2 transcription and stabilizing c-MYC protein in colorectal cancer
Source: J Exp Clin Cancer Res. 2024 Jan 2;43:7. doi: 10.1186/s13046-023-02873-0 (PMC10759692; doi:10.1186/s13046-023-02873-0)
Supplement: Supplementary file 1 — Additional file 1: Fig. S1. EFTUD2 is significantly upregulated in 5-FU chemotherapy-resistant cells of CRC, related to Fig. 1. A Analysis of the differential expression genes (DEGs) expression in 5-FU resistant CRC cell lines compared with parental cell lines using three GEO datasets (GSE166900, GSE81005, GSE81008). B Analysis of 18 significantly upregulated genes shared among CRC chemoresistant cells using Venn diagram. C Multivariate Cox regression analysis of 18 candidate genes and identification of top four genes (EFTUD2, VIM, EFNB2, and PLAC1) using TCGA. D Analysis of EFTUD2 expression in the non-responsive group compared with response group to FOLFOX and FOLFIRI using CTRP. E GSEA using the gene set linked to EFTUD2 in CRC from TCGA. **P < 0.01. Fig. S2. High EFTUD2 expression is a potential independent predictor of poor prognosis in CRC, related to Fig. 2. A Analysis of EFTUD2 in predicting survival probability in CRC patients based on Cox regression analysis using Nomogram-related model. B Calibration curve analysis depicting the differentiation between predicted and actual survival rates of the model at different time points (1-year, 2-year, and 3-year). Fig. S3. EFTUD2 attenuates the chemotherapy efficacy of 5-FU in vitro, related to Fig. 3. A MTT assay showing the influence of various concentrations of 5-FU on viability of SW480, HCT116, and Caco-2 cells. B Clone formation and EdU assays showing the influence of EFTUD2 upregulation on cell survival and proliferation in SW480 cells treated with 5-FU. C and D TUNEL apoptosis assays showing the influence of EFTUD2 modulation on apoptosis in SW480, HCT116, and Caco-2 cells treated with 5-FU. Each bar represents the mean values ± SD, *P < 0.05; **P < 0.01; ***P < 0.001. Fig. S4. Correlation and expression analysis of EFTUD2 with candidate genes, related to Fig. 5. A Heat map showing the top five genes correlated with EFTUD2 in CRC using TCGA. B-F Differentiation expression analysis showing the mRNA expressio [file 13046_2023_2873_MOESM1_ESM.docx]

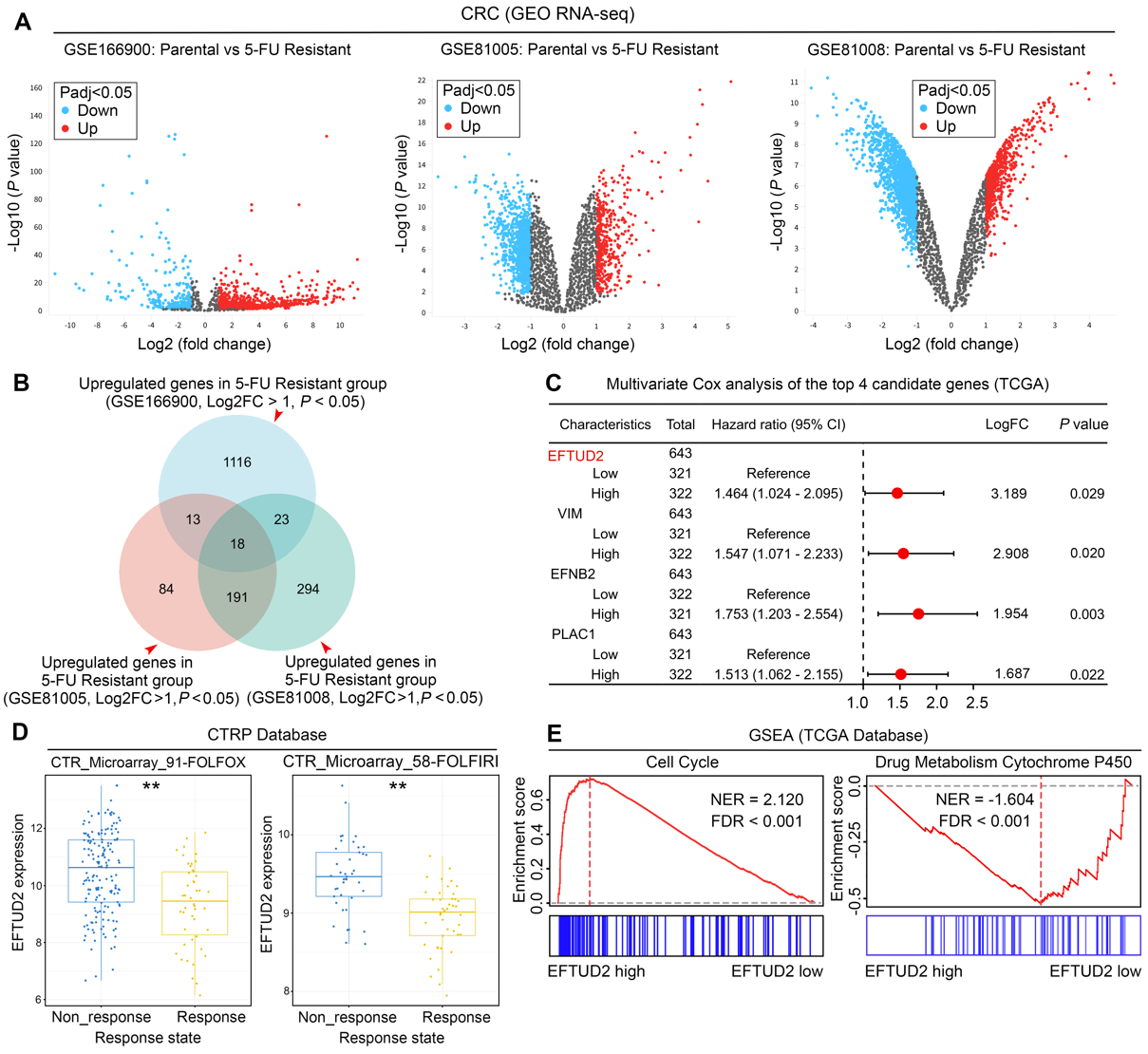


**Fig. S1 EFTUD2 is significantly upregulated in 5-FU chemotherapy-resistant cells of CRC, related to Fig. 1. A** Analysis of the differential expression genes (DEGs) expression in 5-FU resistant CRC cell lines compared with parental cell lines using three GEO datasets (GSE166900, GSE81005, GSE81008). **B** Analysis of 18 significantly upregulated genes shared among CRC chemoresistant cells using Venn diagram. **C** Multivariate Cox regression analysis of 18 candidate genes and identification of top four genes (EFTUD2, VIM, EFNB2, and PLAC1) using TCGA. **D** Analysis of EFTUD2 expression in the non-responsive group compared with response group to FOLFOX and FOLFIRI using CTRP. **E** GSEA using the gene set linked to EFTUD2 in CRC from TCGA. ***P* < 0.01.


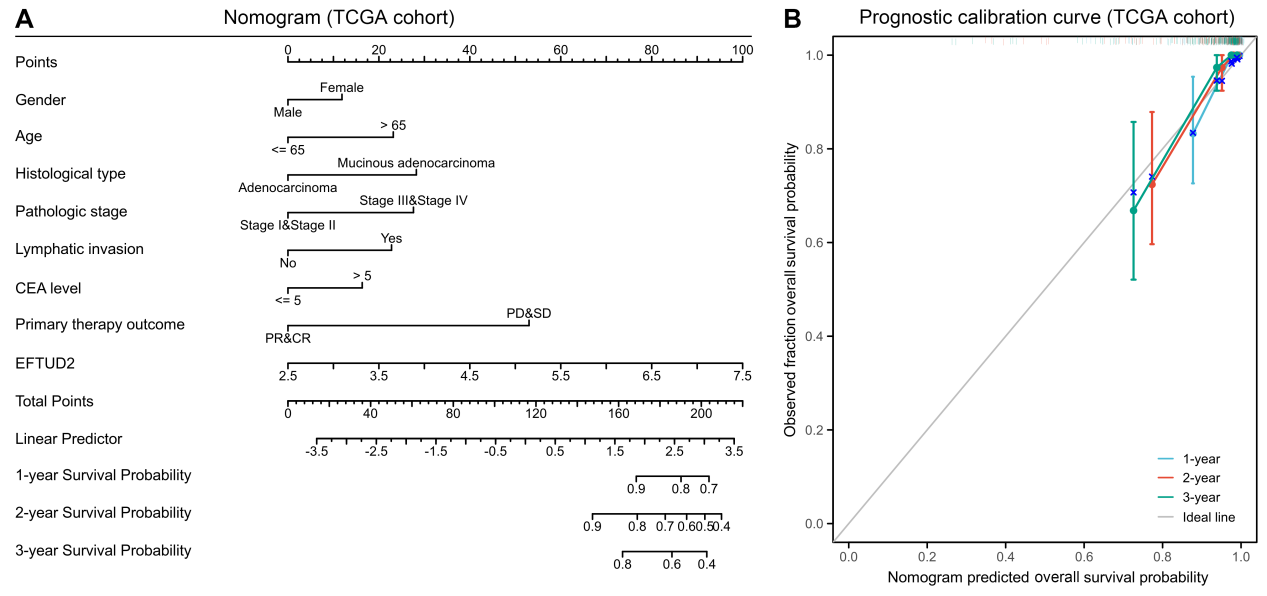


**Fig. S2 High EFTUD2 expression is a potential independent predictor of poor prognosis in CRC, related to Fig. 2. A** Analysis of EFTUD2 in predicting survival probability in CRC patients based on Cox regression analysis using Nomogram-related model. **B** Calibration curve analysis depicting the differentiation between predicted and actual survival rates of the model at different time points (1 year, 2 year, and 3 year).


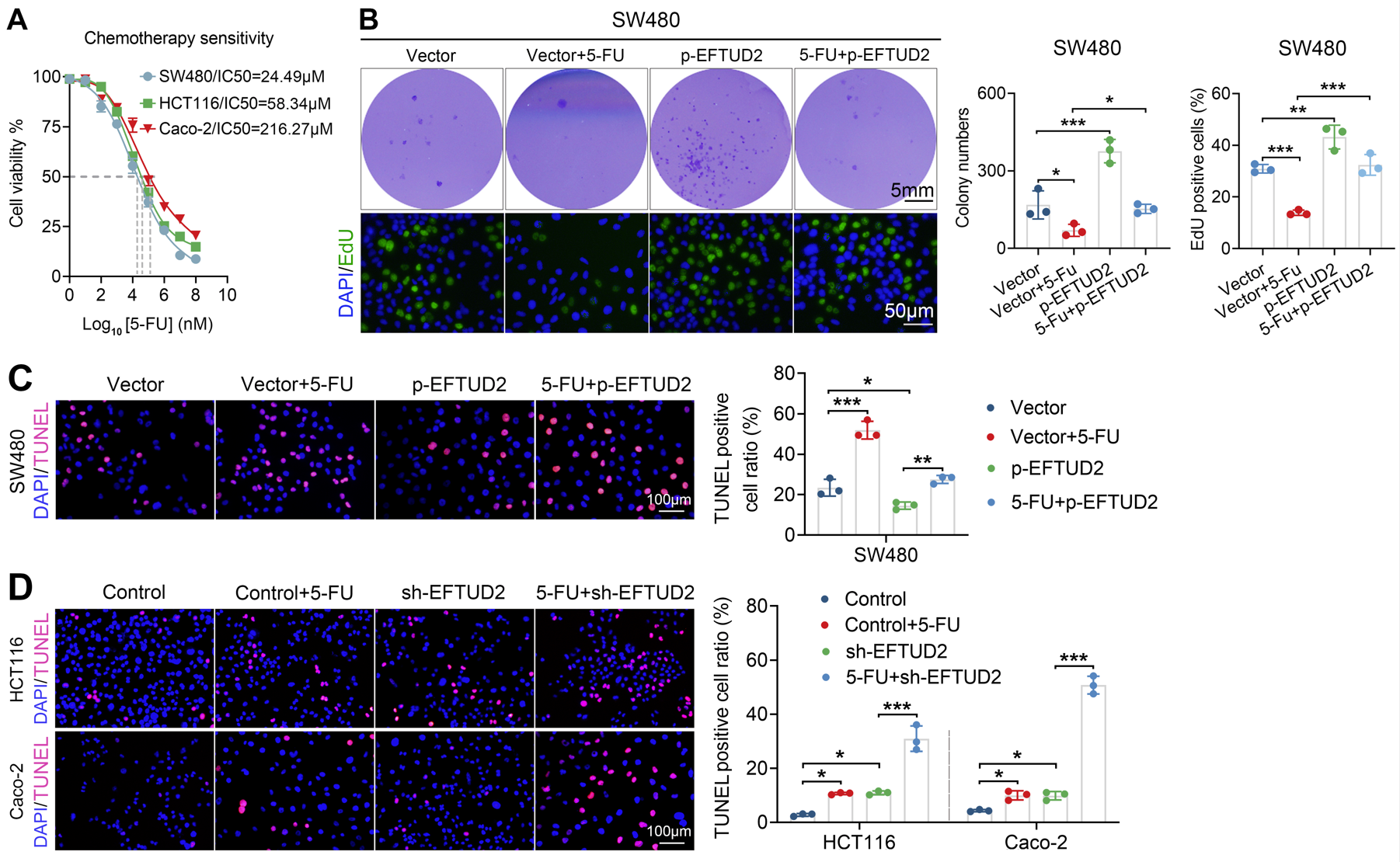


**Fig. S3 EFTUD2 attenuates the chemotherapy efficacy of 5-FU *in vitro*, related to Fig. 3. A** MTT assay showing the influence of various concentrations of 5-FU on viability of SW480, HCT116, and Caco-2 cells. **B** Clone formation and EdU assays showing the influence of EFTUD2 upregulation on cell survival and proliferation in SW480 cells treated with 5-FU. **C and D** TUNEL apoptosis assays showing the influence of EFTUD2 modulation on apoptosis in SW480, HCT116, and Caco-2 cells treated with 5-FU. Each bar represents the mean values ± SD, **P* < 0.05; ***P* < 0.01; ****P* < 0.001.


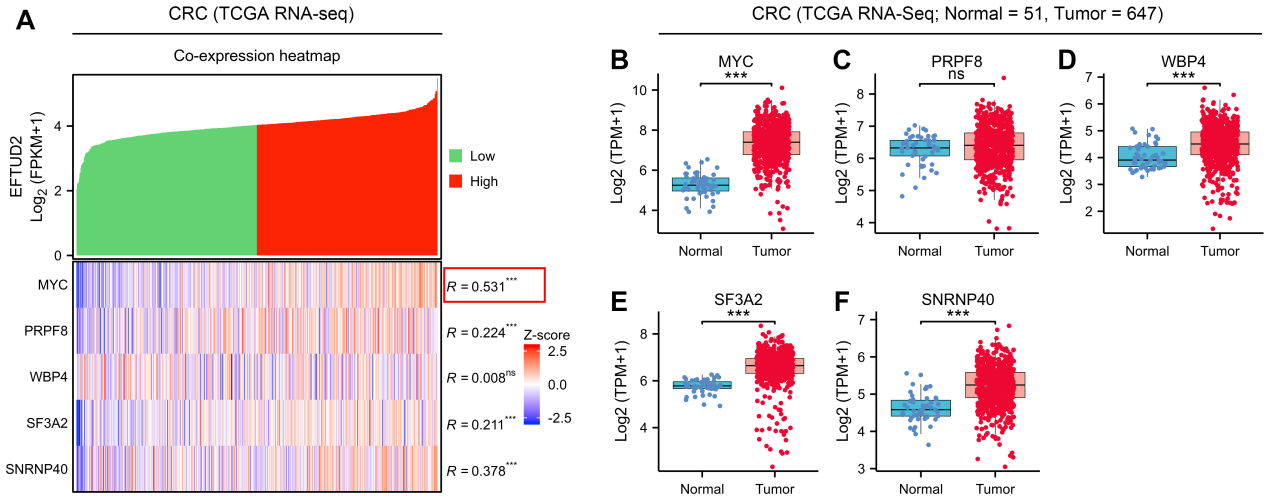


**Fig. S4 Correlation and expression analysis of EFTUD2 with candidate genes, related to Fig. 5. A** Heat map showing the top five genes correlated with EFTUD2 in CRC using TCGA. **B-F** Differentiation expression analysis showing the mRNA expression levels of MYC, PRPF8, WBP4, SF3A2, and SNRNP40 in CRC tissues compared with normal tissues using TCGA. ****P* < 0.001.


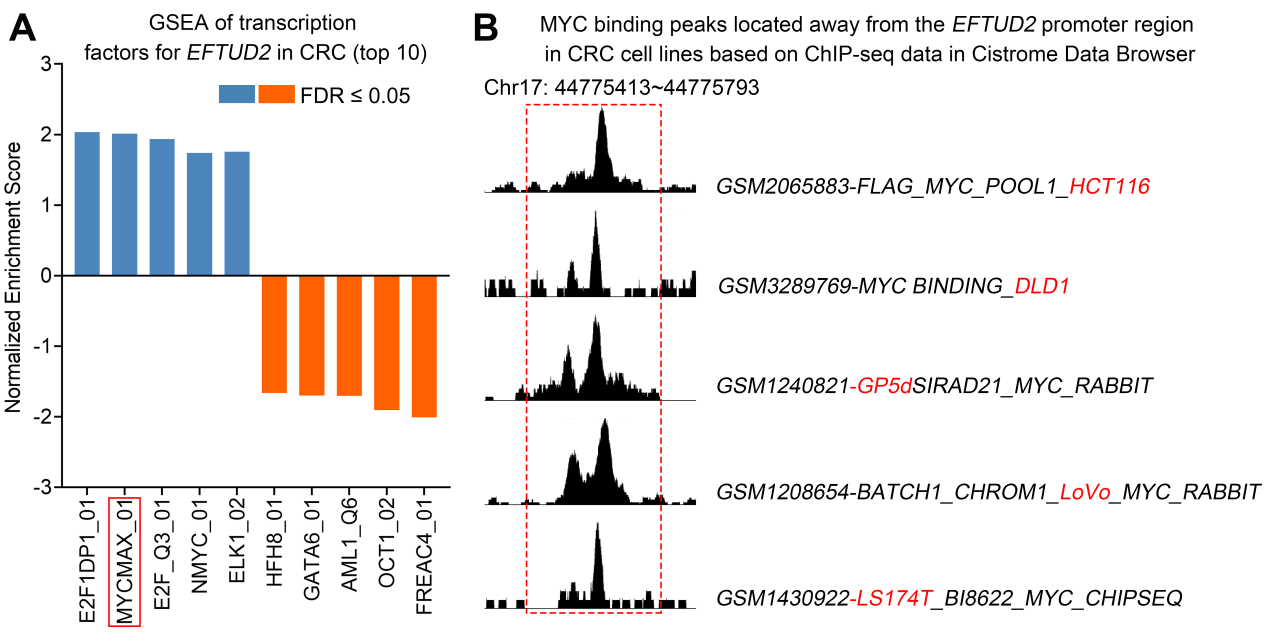


**Fig. S5 MYC promotes the transcription of EFTUD2, related to Fig. 7. A** GSEA showing the transcription factors related to EFTUD2 in CRC using TCGA. **B** ChIP-seq analysis of MYC binding peaks on EFTUD2 using Cistrome Data Browser.


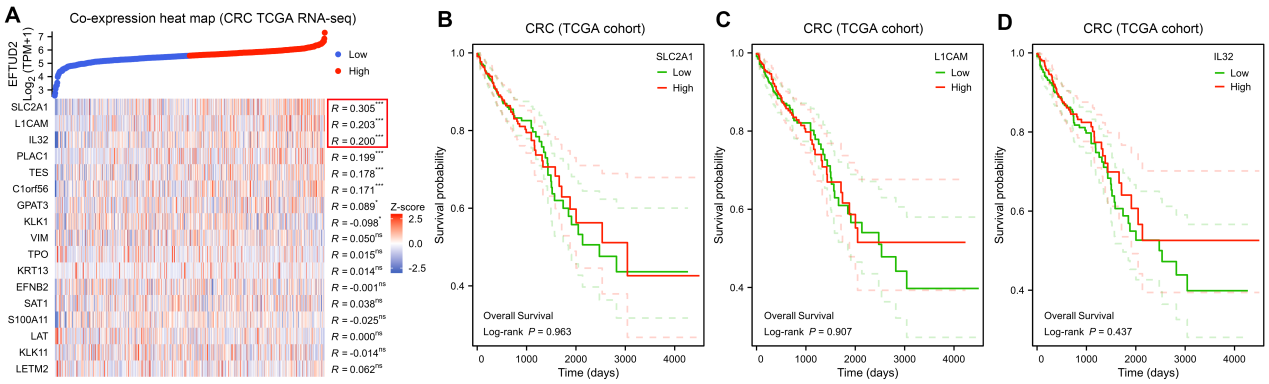


**Fig. S6** **Correlation and overall survival** **analysis of EFTUD2 with 17 significantly upregulated genes shared in CRC chemoresistant cells. A** Heat map showing the correlation between EFTUD2 and 17 significantly upregulated genes using the TCGA. **B** Kaplan-Meier analysis of overall survival in two groups of CRC patients stratified by high and low expression of top three genes (SLC2A1, L1CAM, and IL32) using TCGA.


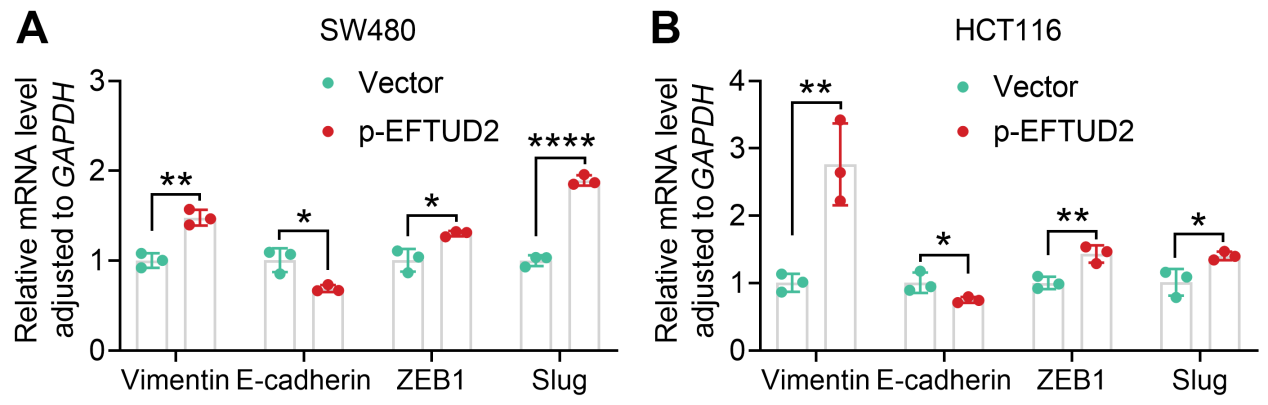


**Fig. S7 Overexpressing EFTUD2 promotes the epithelial-mesenchymal transition (EMT) phenotype. A and B** Analysis of EMT markers (Vimentin, E-cadherin, ZEB1, and Slug) after overexpressing EFTUD2 in SW480 and HCT116 cells using RT-qPCR. Each bar represents the mean values ± SD, **P* < 0.05; ***P* < 0.01; *****P* < 0.0001.

Supplementary Table 1. Correlation analyses of EFTUD2 with sensitivity to common chemotherapeutic drugs in CRC

| **Compound** | **RNAmolecule** | **Omics** | **Correlation R** | **Spearman. FDR** |
| --- | --- | --- | --- | --- |
| 5-Fluorouracil | EFTUD2 | Expression | -0.418 | *P* = 0.004 |
| Capecitabine | EFTUD2 | Expression | -0.420 | *P* < 0.001 |
| Cisplatin | EFTUD2 | Expression | -0.363 | *P* = 0.007 |
| Irinotecan | EFTUD2 | Expression | -0.406 | *P* < 0.001 |
| Oxaliplatin | EFTUD2 | Expression | -0.283 | *P* < 0.001 |

Supplementary Table 2. Correlation analyses **between EFTUD2 expression and clinicopathological characteristics of CRC patients using TCGA**

| **Characteristics** | **Low expression of EFTUD2** | **High expression of EFTUD2** | ***P* value** |
| --- | --- | --- | --- |
| n | 322 | 322 |  |
| OS event, n (%) |  |  | **0.004** |
| Alive | 272 (42.2%) | 243 (37.7%) |  |
| Dead | 50 (7.8%) | 79 (12.3%) |  |
| Anatomic neoplasm subdivision, n (%) |  |  | 0.514 |
| Ascending Colon&Descending Colon | 55 (13.1%) | 53 (12.6%) |  |
| Rectum&Sigmoid Colon&Transverse Colon | 148 (35.2%) | 165 (39.2%) |  |
| Primary therapy outcome, n (%) |  |  | **0.004** |
| PD&SD | 10 (4.4%) | 28(8.9%) |  |
| PR&CR | 141 (45.2%) | 133 (42.6%) |  |
| Histological type, n (%) |  |  | **0.015** |
| Adenocarcinoma | 266 (42%) | 284 (44.9%) |  |
| Mucinous adenocarcinoma | 52 (8.2%) | 31 (4.9%) |  |
| Pathologic stage, n (%) |  |  | **0.003** |
| Stage I&Stage II | 175 (28.1%) | 138 (22.2%) |  |
| Stage III&Stage IV | 136 (21.8%) | 174 (27.9%) |  |
| Gender, n (%) |  |  | 0.580 |
| Female | 147 (22.8%) | 154 (23.9%) |  |
| Male | 175 (27.2%) | 168 (26.1%) |  |
| Age, n (%) |  |  | **0.017** |
| <= 65 | 123 (19.1%) | 153 (23.8%) |  |
| > 65 | 199 (30.9%) | 169 (26.2%) |  |
| Pathologic T stage, n (%) |  |  | 0.970 |
| T1&T2 | 65 (10.1%) | 66 (10.3%) |  |
| T3&T4 | 254 (39.6%) | 256 (39.9%) |  |
| Pathologic N stage, n (%) |  |  | 0.873 |
| N0 | 183 (28.6%) | 185 (28.9%) |  |
| N1&N2 | 137 (21.4%) | 135 (21.1%) |  |
| Pathologic M stage, n (%) |  |  | 0.440 |
| M0 | 240 (42.6%) | 235 (41.7%) |  |
| M1 | 41 (7.3%) | 48 (8.5%) |  |
| Lymphatic invasion, n (%) |  |  | 0.785 |
| No | 177 (30.4%) | 173 (29.7%) |  |
| Yes | 120 (20.6%) | 112 (19.2%) |  |
| CEA level, n (%) |  |  | 0.456 |
| <= 5 | 124 (29.9%) | 137 (33%) |  |
| > 5 | 79 (19%) | 75 (18.1%) |  |

| Supplementary Table 3. The sequence of primers used in Fig.7K and Fig. 8 | |
| --- | --- |
| **Primers** | **Sequence (5′-3′)** |
| p-c-MYC Forward: | 5′-TAAGCTTGGTACCGAGCTCGGATCCCTGGATTTTTTTCGGGTAGTGGAAAACCAGCC-3′ |
| p-c-MYC Reverse: | 5′-GATGGAAGGGCCCTCTAGACTCGAGCGCACAAGAGTTCCGTAGCTGTTC-3′ |
| sh-c-MYC#1: | 5′-UUGAGGGGCAUCGUCGCGGG-3′ |
| sh-c-MYC#2: | 5′-GCUGCACCGAGUCGUAGUCG-3′ |
| EFTUD2-promoter-WT Forward: | 5′-TAACCTCTGAAAGAGGAACTTGGTTAGGTACCATGTGTCAGACACTTGCTCAGTCT-3′ |
| EFTUD2-promoter-WT Forward: | 5′-TCGTACACCTTGGAAGCCATGGTGGCTAGCGATGCTCTCGCCTGCTCAGCT-3′ |
| EFTUD2-promoter-Mut Forward: | 5′-CTAGTGAGTTGAGCTAGATTTTGTGGCGGGTGCATATCTGAGGCTG-3′ |
| EFTUD2-promoter-Mut Forward: | 5′-CACTCTGGACATCATGTCACTCAGATTGGGAGGTGTTACAAAAATCTG-3′ |
